# Layer 5 Intratelencephalic Neurons in the Motor Cortex Stably Encode Skilled Movement

**DOI:** 10.1523/JNEUROSCI.0428-23.2023

**Published:** 2023-10-25

**Authors:** Takanori Shinotsuka, Yasuhiro R. Tanaka, Shin-Ichiro Terada, Natsuki Hatano, Masanori Matsuzaki

**Affiliations:** ^1^Department of Physiology, Graduate School of Medicine, University of Tokyo, Tokyo 113-0033, Japan; ^2^Brain Science Institute, Tamagawa University, Machida, Tokyo 194-8610, Japan; ^3^International Research Center for Neurointelligence, University of Tokyo Institutes for Advanced Study, Tokyo 113-0033, Japan; ^4^Brain Functional Dynamics Collaboration Laboratory, RIKEN Center for Brain Science, Saitama 351-0198, Japan

**Keywords:** encoding/decoding, intratelencephalic neurons, motor cortex, motor learning, skilled forelimb movement, two-photon imaging

## Abstract

The primary motor cortex (M1) and the dorsal striatum play a critical role in motor learning and the retention of learned behaviors. Motor representations of corticostriatal ensembles emerge during motor learning. In the coordinated reorganization of M1 and the dorsal striatum for motor learning, layer 5a (L5a) which connects M1 to the ipsilateral and contralateral dorsal striatum, should be a key layer. Although M1 L5a neurons represent movement-related activity in the late stage of learning, it is unclear whether the activity is retained as a memory engram. Here, using *Tlx3-Cre* male transgenic mice, we conducted two-photon calcium imaging of striatum-projecting L5a intratelencephalic (IT) neurons in forelimb M1 during late sessions of a self-initiated lever-pull task and in sessions after 6 d of nontraining following the late sessions. We found that trained male animals exhibited stable motor performance before and after the nontraining days. At the same time, we found that M1 L5a IT neurons strongly represented the well-learned forelimb movement but not uninstructed orofacial movements. A subset of M1 L5a IT neurons consistently coded the well-learned forelimb movement before and after the nontraining days. Inactivation of M1 IT neurons after learning impaired task performance when the lever was made heavier or when the target range of the pull distance was narrowed. These results suggest that a subset of M1 L5a IT neurons continuously represent skilled movement after learning and serve to fine-tune the kinematics of well-learned movement.

**SIGNIFICANCE STATEMENT** Motor memory persists even when it is not used for a while. IT neurons in L5a of the M1 gradually come to represent skilled forelimb movements during motor learning. However, it remains to be determined whether these changes persist over a long period and how these neurons contribute to skilled movements. Here, we show that a subset of M1 L5a IT neurons retain information for skilled forelimb movements even after nontraining days. Furthermore, suppressing the activity of these neurons during skilled forelimb movements impaired behavioral stability and adaptability. Our results suggest the importance of M1 L5a IT neurons for tuning skilled forelimb movements over a long period.

## Introduction

The learning and execution of motor skills are essential for life. Once we acquire a specific movement, we can reproduce it even after not performing it for a long time (Arthur et al., 1998; Hikosaka et al., 2002b), which inevitably requires the formation of motor memory. Motor memory is thought to be stored across cortical and subcortical brain areas (Hikosaka et al., 2002a; Doyon et al., 2003), with the primary motor cortex (M1) and dorsal striatum playing particularly critical roles in this learning (Penhune and Doyon, 2002; Yin et al., 2009; Galea et al., 2011; Orban de Xivry et al., 2011; Santos et al., 2015). In mice, synaptic plasticity and reorganization of task-related activity occur in M1 and the dorsal striatum during learning of a variety of forelimb motor tasks, such as the reaching task, lever-pull/push task, and rotarod task (Xu et al., 2009; Masamizu et al., 2014; Peters et al., 2014; Hayashi-Takagi et al., 2015; Santos et al., 2015; Lopez-Huerta et al., 2021). Inhibition of synaptic plasticity in M1 or striatum impairs this motor learning (Yin et al., 2009; Hayashi-Takagi et al., 2015). Dorsolateral striatum (DLS), which receives strong projections from M1 and returns the outputs to M1 through downstream pathways including the thalamus (Aoki et al., 2019), is crucial for reorganization in the late phase of learning (Costa et al., 2004; Yin et al., 2009; Smith and Graybiel, 2013; Badreddine et al., 2022). The prominent plasticity of the DLS in the late phase may reflect the hierarchy of learning; motor skill learning follows learning of the association between the action and outcome (Hikosaka et al., 2013).

In the coordinated reorganization of M1 and DLS during motor skill learning, the upper sublayer of layer 5 (L5a) in M1 should be a key sublayer because it contains many intratelencephalic (IT) neurons that connect M1 with ipsilateral and contralateral DLS (Winnubst et al., 2019). We previously found that M1 L5a neurons and retrogradely labeled L5a IT (or crossed corticostriatal; Morishima and Kawaguchi, 2006) neurons increased motor information from the middle stage of learning a self-initiated forelimb lever-pull task (Masamizu et al., 2014). Layer 5 (L5) IT neurons are known to well represent the kinematics of forelimb movements (Currie et al., 2022; Park et al., 2022). Furthermore, it is assumed that the DLS continuously integrates task-relevant information to constrain the execution of motor habits after learning (Rueda-Orozco and Robbe, 2015). Striatal postsynaptic sites that receive inputs from reactivated M1 neurons showed increased excitatory postsynaptic currents after a rest period following learning (Hwang et al., 2022). Consistent with this, transient inactivation of M1 L5a IT neurons impairs motor performance (Park et al., 2022), whereas inhibition of corticostriatal plasticity increases trial-to-trial variability in movement (Santos et al., 2015). Thus, M1 IT neuron activity and propagation of this activity to the DLS may be required for the precise execution of well-learned movement. However, it remains unclear whether the motor-related activity of single neurons is stable or not in the motor cortex (Carmena et al., 2005; Chestek et al., 2007; Hwang et al., 2019).

Here, we examined whether M1 L5a IT neurons maintained task-relevant motor information over nontraining days after the late phase of learning. Using head-fixed *Tlx3-Cre* mice to probe L5a IT neurons, which project their axons to the dorsal striatum (Gerfen et al., 2013), we simultaneously conducted two-photon calcium imaging during a lever-pull task in the forelimb M1 [caudal forelimb area (CFA)], and video tracked forelimb and orofacial movements (Hira et al., 2013; Masamizu et al., 2014; Terada et al., 2022). Then, constructing an encoding model, we assessed whether ensembles of CFA L5a IT neurons specifically represented the skilled forelimb movement but not the accompanying uninstructed movements. We also performed Cre-dependent optogenetic perturbation to reveal that M1 L5a IT neurons were important for performing the forelimb movement with high reproducibility when the lever-pull task required fine adjustments.

## Materials and Methods

### Animals

All animal procedures were approved by the Animal Care and Use Committees of the University of Tokyo Graduate School of Medicine and followed the care and use guidelines of the institute. Mice (male, 2–4 months old unless otherwise stated) were housed under a 12 h light/dark cycle (light cycle, 8:00 A.M.–8:00 P.M.) with access to food and water *ad libitum*. Two mouse lines were used, wild-type (C57BL/6JJmsSlc, obtained from Japan SLC) and Tlx3-Cre [B6.FVB(Cg)-Tg(Tlx3-cre)PL56Gsat/Mmucd (RRID:MMRRC_041158-UCD)]. The *Tlx3-Cre* mouse line has *Cre*-expressing neurons in a subpopulation of IT neurons in L5a and superficial layer 5b (Gerfen et al., 2013; Callaway et al., 2021; Muñoz-Castañeda et al., 2021; Im et al., 2022). Virtually all *Tlx3-Cre*-positive (*Tlx3*+) neurons have their axonal arborizations confined within the telencephalon and have few axons in the thalamus, brainstem, and spinal cord (Gerfen et al., 2013; Callaway et al., 2021; Im et al., 2022). In addition, the majority of L5a IT neurons in the secondary motor cortex innervate the striatum (Im et al., 2022). We thus consider that *Tlx3*+ neurons well represent a subset of L5a IT neurons that project to the striatum.

### Surgery for head plate attachment

The mice received intraperitoneal injections of dexamethasone (1.32 mg/kg body weight), carprofen (6 mg/kg), sulfadiazine (24 mg/kg), and trimethoprim (4.8 mg/kg) for preoperative treatment. Mice were anesthetized with an intraperitoneal injection of ketamine (74 mg/kg) and xylazine (10 mg/kg) and were placed in a stereotaxic frame with ear bars (Narishige). Their eyes were covered with eye ointment (Tarivid, Santen Pharmaceutical) to keep them moist. The scalp overlaying the neocortex was incised and the skull was cleaned. A custom head plate (Tsukasa Giken; Hira et al., 2013) was then attached to the skull with dental cement (Fuji lute BC, GC; Bistite II or EsteCem II, Tokuyama Dental), and the skull was covered with a dental acrylic resin (Super-Bond, Sun Medical). After surgery, mice were individually housed and allowed to recover for at least 3 d.

### Self-initiated lever-pull task

The self-initiated lever-pull task proceeded in a similar way to that previously described (Hira et al., 2013; Masamizu et al., 2014; Tanaka et al., 2018). Mice were placed in an immovable stainless steel body holder tube and head fixed in the task device. A lever was placed in front of the right forelimb (the natural position), and a constant magnetic force of 0.03 N or 0.05 N was applied to the lever to ensure it returned to the natural position. Head-fixed mice were rewarded when they pulled the lever past a threshold for 0.6 s. The threshold was set at 2.5 mm from the natural position. In this task, the lever pulling was limited to 5.0 mm from the natural position, and the mice obtained a reward even when they applied excessive pulling force. A drop of water reward (4 µl) was delivered to the spout tip after a delay of ∼176 ms after a reward signal command from a National Instruments Data Acquisition (NIDAQ) system (Tanaka et al., 2018; Terada et al., 2022). To initiate a new trial, mice had to return the lever and maintain it between the threshold and its natural position for more than a certain period (the set wait duration). The set wait duration was gradually increased across the values 0.01, 0.2, 0.4, 0.6, and 1.0 s every 25 successful trials throughout the sessions, except for the two-photon imaging experiments. Failure trials were defined as pulling attempts in which the lever was returned, even briefly, between the threshold and its natural position when required to be pulled past the threshold for 0.6 s.

Drinking water was restricted to 1 ml/day from at least 4 d before the learning sessions, and the mice were maintained at 80–85% of their normal body weight during the task sessions. Mice were acclimated to head fixation in the body holder in the task device for 3 d in advance of the learning sessions. They then performed a 30–60 min task session per day for 5–6 d per week. On days without a task session, mice were maintained on water (1 ml/day) or water agar chunks (1 g/day). After 14 learning sessions, the mice were individually housed for 6 d of nontraining under water restriction (1 ml/day), after which they were retrained in two test sessions. The task device was controlled by a program written with LabVIEW software (National Instruments).

### Target-zone lever-pull task

A target-zone lever-pull task was instigated, in which the aim was to train mice to continuously hold the lever within a set range (target zone) for a set period (*T*_target_). Mice were head fixed in a slightly modified version of the apparatus used in the self-initiated lever-pull task. Removal of a stopper that restricted the range of the lever in the pulling direction allowed the lever to be pulled >10 mm. The magnetic force was constantly applied to return the lever to its natural position. Each trial consisted of five task states, which were intertrial interval (ITI), Ready, Go, Success, and Failure. The task state moved from ITI to Ready by holding the lever below a threshold of 0.5 mm from the natural position for 1 s. Any lever-pull movement exceeding the threshold during the ITI reset the holding time to zero and extended the ITI. During the Ready state, pulling the lever past the threshold moved the task state to Go, and the mouse was given a 1 s response period. During the response period, if the lever was continuously in the target zone for *T*_target_, a 4 µl drop of water was delivered immediately, and the task state shifted to Success. However, if the trial was not successful within the response period, the task state was changed to Failure. Both Success and Failure states lasted for 2 s, and any lever pulls during these states were ignored. The task state then returned to ITI. The target zone was varied according to the number of successes from the start of each learning session. The target zone started with a range of ±3.5 mm with the center at 5 mm distance from the natural position in the pulling direction. The range was decreased according to the formula ±*k^n^* × 3.5 mm, where *k* = 0.995 and *n* = cumulative number of successes in a session. In addition, when the time between the last reward and the next lever-pull trial (*T*_wait_ s) was long, we considered that the task was difficult for the mice and added *T*_wait_ × 0.004 mm to the range. During the Go state, a 10 kHz pure tone was presented while the lever was in the target zone to provide sensory feedback on the location of the target zone. *T*_target_ was set at 100 ms for the first and second sessions and at 200 ms for the subsequent sessions. After 14 learning sessions, the mice performed an additional session with the target range fixed at 5 ± 2.5 mm from the natural position, with this being followed by the optogenetic experiment.

### Pharmacology for NMDA receptor blockade

For NMDA receptor (NMDAR) blockade experiments, MK-801 (0.25 mg/kg; catalog #M107, Sigma-Aldrich) was dissolved in saline solution (0.9% NaCl solution) and injected intraperitoneally into the mice (Zuo et al., 2005). Saline solution was injected as a control. Saline solution or MK-801 solution was injected into mice 20 min before and after each session according to the following schedules: the first to third learning sessions, saline; the fourth to 14th learning sessions, saline or MK-801; the first and second test sessions, none or saline. For the experiments with MK-801 administration in the first test session, mice were trained in the self-initiated lever-pull task without injection and were administered MK-801 solution 20 min before and after the first test session.

The absolute trajectory error was defined as the mean absolute difference between the lever trajectory and the reference expert trajectory (average lever trajectory of successful trials in the 13th and 14th learning sessions in the group of saline-administered control mice) during the first 0.6 s after the lever-pull initiation.

### Surgery for two-photon calcium imaging

Dexamethasone (1.32 mg/kg), carprofen (6 mg/kg), sulfadiazine (24 mg/kg), and trimethoprim (4.8 mg/kg) were injected into mice intraperitoneally for preoperative treatment. Mice were anesthetized with an intraperitoneal injection of ketamine (74 mg/kg) and xylazine (10 mg/kg) or inhalation of isoflurane (1–4%), and were placed in a stereotaxic frame. A square craniotomy of ∼0.5 × 0.5 mm was prepared in the left hemisphere. A quartz-glass pipette was pulled (P-2000, Sutter Instrument) and beveled to a tip of 20–35 µm diameter. The pipette was backfilled with mineral oil and connected to a Nanoject III injector (Drummond Scientific). The virus mixture [adeno-associated virus (AAV)5-CAG-GFP (1–7 × 10^12^ vector genomes ml^−1^) and AAV1-Syn-flex-NES-jRGECO1a (3.0 × 10^12^ vector genomes ml^−1^), both obtained from Addgene] was front loaded into the pipette. The virus solution was injected into the left CFA of *Tlx3-Cre* mice according to the following coordinates from the bregma: 0.5 mm anterior, 1.0 mm lateral, 0.5 mm ventral; 0.5 mm anterior, 1.4 mm lateral, 0.5 mm ventral; 0.1 mm anterior, 1.2 mm lateral, 0.5 mm ventral (20–40 nl per site). The pipette was left for 5 min after each injection to prevent backflow. The craniotomy was covered with dental silicone (KwikCast, World Precision Instruments), and the skin was sutured. Mice were allowed to recover for 1–3 weeks. After recovery, a craniotomy (∼2.5 mm diameter) was performed centered at 0.2 mm anterior and 1.2 mm lateral from the bregma. A stainless steel tube (2.0 mm outer diameter; 1.9 mm inner diameter; 0.5 mm length) covered at one end with a glass coverslip (0.13–0.17 mm thickness, 2.0 mm diameter; Matsunami Glass; Hasegawa et al., 2020) was attached onto the exposed dura and fixed to the skull with cyanoacrylate glue (Vetbond, 3M) and dental acrylic resin (SuperBond). A head plate was attached to the skull as described above, and mice were allowed to recover in their home cages for at least 5 d.

### Two-photon calcium imaging

Images were acquired using an FVMPE-RS system (Olympus) with a femtosecond laser (InSight DS+ dual, Spectra-Physics) tuned to 1020–1040 nm. The objective was a 25× water immersion lens [working distance, 2 mm; numerical aperture (NA), 1.05; XLPLN25XWMP2, Olympus]. Fluorescence signals were collected with gallium arsenide phosphide (GaAsP) photomultiplier tubes (Hamamatsu Photonics) after bandpass filtering (495–540 nm for GFP and 575–645 nm for jRGECO1a; FV30-FGR, Olympus).

Mice were head fixed in the task setup under the objective lens, and their body was rotated at angles of 0°–3° by a two-axis goniometer so that the cranial window was flat to the objective lens. A series of 54,000 continuous images [field of view (FOV), 512 × 512 pixels, 509.1 µm × 509.1 µm] was acquired at 30 Hz. The imaging area was centered at 1.2 mm lateral and 0.2 mm anterior from the bregma. The focal planes were located 445.11 ± 17.72 µm (*n* = 6 mice) below the brain surface. To align to the same imaging plane of each mouse, images of superficial vessels and cells expressing GFP were used as reference images. Imaging experiments were performed during the 13th and 14th learning sessions (LS13 and LS14 hereafter, respectively) as well as the first and second test sessions (TS1 and TS2 hereafter, respectively).

In the learning sessions for the two-photon imaging experiment, the set wait duration was gradually increased from 0.01 to 1 s for every two consecutive sessions with >150 successful trials (Tanaka et al., 2018). The set wait duration was fixed for each animal in the imaging experiment sessions. The set wait duration in the imaging experiment was 0.60 ± 0.14 s (*n* = 6 mice).

### Video acquisition of body movements

Movements of animals were recorded as previously described (Terada et al., 2022). In brief, two synchronized high-speed cameras (Scout scA640-70gm, Basler), each of which was fitted with a fixed focus lens (M0814-MP2, CBC), were positioned to record the bodies (bottom) and faces (side) of the mice. The images were acquired at 70 Hz.

### Surgery for optogenetics

Virus injection was performed as described above, and pAAV-CKIIa-stGtACR1-FusionRed (Addgene viral plasmid #105679-AAV5, http://n2t.net/addgene:105679; RRID:Addgene_105679) and pAAV_hSyn1-SIO-stGtACR1-FusionRed (Addgene viral plasmid #105678-AAV5, http://n2t.net/addgene:105678; RRID:Addgene_105678) were gifts from Ofer Yizhar (Mahn et al., 2018). For the optogenetics experiments in the self-initiated lever-pull task, the virus solution AAV5-CaMKII-stGtACR1-fusionRed (7.0 × 10^12^ vector genomes ml^−1^) or AAV5-Syn-SIO-stGtACR1-FusionRed (7.0 × 10^12^ vector genomes ml^−1^) was front loaded into a quartz-glass pipette (20–30 µm tip diameter) connected to a Nanoject III. The virus solution was injected into the left CFA of wild-type or *Tlx3-Cre* mice 0.6 mm ventral from the dura, centered at 0 mm anterior and 1.25 mm lateral from the bregma (100 or 200 nl per site, two or four sites, total 400 nl). A small and thick glass coverslip (0.25–0.35 mm thickness, 2.0 mm diameter; Matsunami Glass) was bonded to a larger thin glass coverslip (0.08–0.12 mm thickness, 3.5 mm diameter; Matsunami Glass) with an ultraviolet curable optical glue (NOA 61, Norland Products; Tanaka et al., 2018), and this double-layered glass was fixed to the skull with cyanoacrylate glue (Vetbond) and dental acrylic resin (SuperBond).

For the experiments with the target-zone lever-pull task, AAV5-Syn-SIO-stGtACR1-FusionRed (2.1 × 10^13^ vector genomes ml^−1^) was front loaded into a quartz-glass pipette (20–30 µm tip diameter) connected to a 5 µl Hamilton syringe (87930). The virus solution was injected into the left M1 of *Tlx3-Cre* mice at the following coordinates from the bregma: 0.5 mm anterior, 1.2 mm lateral, 0.6 mm ventral; 0.5 mm anterior, 1.7 mm lateral, 0.6 mm ventral (120 nl per site, total 240 nl). The double-layer glass window was constructed by bonding a 3 × 3 mm rectangular glass coverslip (0.08–0.12 mm thickness; Matsunami Glass) to a 2 × 2 mm rectangular glass coverslip (0.45–0.60 mm thickness; Matsunami Glass) with a UV-curing optical adhesive. The double-layered glass was fixed to the skull with cyanoacrylate glue (Vetbond) and dental acrylic resin (SuperBond). A head plate was attached as described. Mice were allowed to recover for at least 5 d in their home cage.

### *In vivo* optogenetic inactivation

Optogenetic inactivation was modified from a previously described procedure (Kondo and Matsuzaki, 2021; Terada et al., 2022). A 594 nm fiber-coupled laser (OBIS LS 594nm, Coherent) was controlled with laser controller software (OBIS LX/LS single laser remote, Coherent). The light was delivered through an antireflection-coated multimode optical fiber (400–700 nm, diameter 50 mm, NA 0.22; catalog #M50L02S-A, Thorlabs) and a fiber port (f = 7.5 mm, 350–700 nm, diameter 1.23 mm; catalog #PAF2S-7A, Thorlabs) to a two-dimensional galvanometric scanning system (Olympus; Kondo and Matsuzaki, 2021). The scanning laser beam was focused onto the brain surface through an achromatic doublet lens (f = 200 mm; catalog #AC254-200-A-ML, Thorlabs). The laser intensity was set to 6 mW or 12 mW after the lens.

A single 1 s light pulse was triggered when the lever crossed the threshold for the light stimulation (1 mm distant from the natural position), with this being controlled by a NIDAQ system and a pulse generator (STOmk-2, BRC). The jitter between the time of threshold crossing and the onset of the laser irradiation was 4.8 ± 0.9 ms (*n* = 25 trials, mean ± SD). The light was delivered to the left hemisphere in a random selection of 50% of the trials. These photostimulation trials were randomly assigned at equal probability (25% of the total trials) to head plate illumination trials or M1 illumination trials. After each illumination trial, the scanning systems moved to the target for the next stimulation trial. LED lights (590 nm) were placed in front of the mice and were continuously illuminated throughout the session to mask the delivery of the light pulse (Sauerbrei et al., 2020).

In the optogenetic inactivation session of the target-zone lever-pull task, light irradiation was applied continuously for 1 s from the moment the lever first entered the target zone in the Go state. The intensity of the laser was set to 12 mW after the focusing lens. To prevent mice from recognizing the timing of the light irradiation, masking LEDs (orange, blinking at 20 Hz) were placed in front of their eyes to keep them blinking throughout all sessions from the first learning session. The task performances during the first 15 min of the session were assessed using the following five measures: success rate, number of successful trials, minimum width of the target zone within the session, trajectory error from the center of the target zone, and time to get a reward from the first-zone entry. The trajectory error was defined as the absolute value of the mean difference between the lever trajectory and the center of the target zone (5 mm distance from the natural position) during 0–0.2 s after the first target-zone entry.

### *In vivo* electrophysiology

After the optogenetic inactivation experiments, a wild-type mouse that expressed stGtACR1 in CFA was used for the experiment. Before beginning the surgery, dexamethasone (1.32 mg/kg), carprofen (6 mg/kg), sulfadiazine (24 mg/kg), and trimethoprim (4.8 mg/kg) were intraperitoneally injected into the mouse. The mouse was anesthetized with 1.5–2% isoflurane. The glass window was carefully removed from the skull, the dura was removed, and the exposed brain surface was covered with dental silicone (KwikCast) and a thin layer of dental acrylic resin (SuperBond). After 2 h of recovery, the mouse was anesthetized with 1.5–2% isoflurane. The mouse was head fixed in the task setup, and the brain was exposed by carefully removing the covers of dental silicone and dental acrylic resin. A Neuropixels 1.0 probe (Jun et al., 2017, IMEC) coated with CM-DiI (catalog #V22888, Thermo Fisher Scientific) was inserted into the left CFA of the mouse at a 60° angle relative to horizontal using a micromanipulator (SMM-200, Narishige). The reference Ag/AgCl wire, soldered to the probe, was connected to the head plate. The exposed brain surface was submerged in an external solution containing the following (in mm): 140 NaCl, 5 HEPES, 2.5 KCl, 2 CaCl_2_, and 1 MgSO_4_. The probe was allowed to settle for ∼20 min before starting the recording.

Electrical signals were collected from 384 channels at 30 kHz in internal reference mode with the probe tip as the reference and then high-pass filtered (0.3 kHz), amplified at a gain of 250, and digitized in the head stage. The digitized signals were sent to a PC via a PXI interface (PXIe-8381, National Instruments) and were recorded using SpikeGLX software (http://billkarsh.github.io/SpikeGLX/). For the activation of stGtACR1, a single 10 s 594 nm laser light pulse (12 mW at the lens) was generated by a pulse generator (STOmk-2). Six stimulations were performed during the electrophysiological recording. Multielectrode recordings were processed using Kilosort3 (Pachitariu et al., 2023), which corrected the motion drift of the Neuropixels 1.0 probe in the brain and detected spikes from each channel. These spikes were subjected to a clustering algorithm in Kilosort3 and were automatically sorted into many spike clusters. The spike clusters were labeled according to their quality as good, multiunit activity, or noise. We used the good clusters as single units and further manually curated them using the GUI (graphical user interface) of Phy (https://github.com/cortex-lab/phy) to exclude artifact clusters. The channel position of each good cluster along the Neuropixels 1.0 probe was histologically validated in the cortical layer and area according to the channel ID of each spike cluster. We analyzed good clusters located in M1 L5 as M1 L5 units. To normalize the spike rate of each unit, the spike rate during photostimulation was divided by the spike rate for 5 s immediately before each photostimulation onset.

### Simultaneous optogenetic inactivation and two-photon calcium imaging

Two male *Tlx3-Cre* mice (∼10 months old) were used for the experiment. A 240 nl volume of a 1:1 mixture of AAV5-Syn-SIO-stGtACR1-FusionRed (1.1 × 10^13^ vector genomes ml^−1^) and AAV5-Syn-GCaMP6f (1.1 × 10^13^ vector genomes ml^−1^) was injected into left CFA at the following coordinates from the bregma: 0.5 mm anterior, 1.5 mm lateral, and 0.6 mm ventral. The injection site was sealed with a double-layered glass window constructed by bonding a 2 × 4 mm (0.35–0.45 mm thickness) rectangular coverslip to a 3 × 5 mm (0.13–0.17 mm thickness) rectangular coverslip. Approximately 3 weeks postinjection, mice were head fixed in the task setup under the objective lens, as described above.

Modifications were made to the microscope to enable optical stimulation during two-photon calcium imaging. A dichroic mirror (ADM800, Olympus) was inserted between the scanning lens and tube lens. The collimated 594 nm laser (OBIS LS 594nm, Coherent) was introduced to this mirror with Kohler illumination, creating a uniform irradiation spot on the focal plane. The dichroic mirror between the tube lens and the objective lens was changed to a multiband dichroic mirror (FV30-NDMVCOIR, Olympus) with a transmission characteristic of 594 nm. This enabled collection of fluorescence signals with the detector while allowing the 594 nm stimulation light to pass. To cope with contaminating signals from the light illumination during imaging, the optical detector was changed to a silicon photomultiplier (FV30SP-RXSiPM, Olympus), a photon counting device that uses a multipixelized Geiger-mode avalanche photodiode and has photon sensitivity equivalent to a GaAsP photomultiplier tube while being resistant to strong light exposure (Modi et al., 2019). The fluorescence signal from GCaMP6f was detected by the silicon photomultiplier detector after passing through the following optical filters: a bandpass filter (495–540 nm), a dichroic mirror (570 nm cutoff), and a second bandpass filter (495–540 nm; FV30-FGR, Olympus).

Light stimulation was conducted during two-photon calcium imaging performed in awake head-fixed mice. The femtosecond laser for imaging was tuned to 920 nm. A 25× objective lens was used. A series of 40,000 continuous images (FOV size, 254 µm × 254 µm with a digital zoom) was acquired at 30 Hz. The depths of the focal planes from the brain surface were 410, 433, and 450 µm for L5a imaging, and 139 and 170 µm for layer 2/3 (L2/3) imaging. The intensity of the 594 nm laser was 12 mW under the objective lens. To reduce the light illumination artifact affecting the fluorescent signal as much as possible, the light was illuminated during the period when the femtosecond laser was not scanning within the FOV. In the current imaging condition in which the X-line was bidirectionally scanned with a resonant scanner at 8 kHz, the time spent scanning one line was 62.5 µs, and the time from the end of scanning one line within the FOV to the beginning of scanning the next line within the FOV was 25 µs. At the end of scanning each line within the FOV, the horizontal synchronization signal was output from the microscopy controller. This triggered the generation of a pulsed electrical signal from a custom circuit with a monostable multivibrator (74HC423AP, Toshiba), and this signal turned on the 594 nm laser for 15 µs. This 15 µs light illumination was repeated for 1 s in one photostimulation so that the averaged laser intensity during the 1 s period was estimated to be 2.9 mW (12 mW × 15/62.5). This value was almost the same as the intensity of the continuous laser irradiation (3 mW) that was used in our previous study (Terada et al., 2022). The 1 s light stimulation was repeated 100 times at random intervals of 10–15 s.

The radius of the light beam was 0.29 mm at the focal plane (within 10% of the maximum value). This value is ∼1.5 times greater than the radius of the beam diameter used in the *in vivo* optogenetic inactivation conducted in the task-performing mice described above (0.19 mm; Terada et al., 2022). Visible light is strongly scattered even when it enters brain tissue with a sufficiently narrow beam diameter. Because the lateral radius of the area in which neuronal activity is suppressed by stGtACR1 stimulation with a laser with a radius of 0.2 mm was reported to be ∼0.8 mm (Li et al., 2019), we assumed that the lateral radius of the stimulated cortical area would be ∼0.8 mm in our two *in vivo* photostimulation methods.

To confirm the expression of stGtACR1 in each neuron, FusionRed was excited by the femtosecond laser tuned to 1040 nm, and its fluorescence signal was imaged with a red filter (575–645 nm; FV30-FGR, Olympus) after the light stimulation experiment. Regions of interest (ROIs) and time-series calcium traces were extracted using the CaImAn algorithm (Giovannucci et al., 2019). The expression of stGtACR1 in an ROI was visually determined by comparing the images of each ROI cut out with its centroid and the images of FusionRed in the same area.

### Optogenetics inactivation and whole-cell patch-clamp recording in acute slice preparations

Slice preparation and patch-clamp recording were conducted as previously described, with slight modifications (Tanaka et al., 2008; Yamamoto et al., 2020). AAV5-Syn-SIO-stGtACR1-FusionRed (2.1 × 10^13^ vector genomes ml^−1^) virus solution was injected into the left M1 of three *Tlx3-Cre* mice (8 weeks old, two male and one female), as described above. After 3–4 weeks, the mice were anesthetized with an intraperitoneal injection of ketamine (74 mg/kg) and xylazine (10 mg/kg), and were transcardially perfused with ice-cold slicing solution containing the following (in mm): 103 NMDG, 2.5 KCl, 1.2 NaH_2_PO_4_, 10 MgSO_4_, 30 NaHCO_3_, 25 glucose, 20 HEPES, 2 Thiourea, 3 Na-pyruvate,12 *N*-acetyl-l-cysteine, and 0.5 CaCl_2_, with adjustment to pH 7.3–7.4 with HCl and saturation with 95% O_2_ and 5% CO_2_ gas. After decapitation, 300-µm-thick coronal forelimb M1 slices were prepared using a Leica VT 1200 S Microtome and the ice-cold slicing solution, bubbled with 95% O_2_ and 5% CO_2_ gas. Then, cortical slices were placed for 10 min in the slicing solution that was prewarmed at 33°C and bubbled with 95% O_2_ and 5% CO_2_. Slices were kept at room temperature for at least 60 min before experiments in the normal ACSF containing the following (in mm): 125 NaCl, 25 NaHCO_3_, 10 glucose, 2.5 KCl, 1.25 NaH_2_PO_4_, 2 CaCl_2_, and 1 MgCl_2_, bubbled with 95% O_2_ and 5% CO_2_ gas.

The slices were placed in a recording chamber on the stage of a microscope (FV1000, Olympus) with a CMOS (complimentary metal-oxide-semiconductor) camera (ORCA-spark, Hamamatsu Photonics). The oxygenized ACSF was continuously perfused to the recording chamber during recording. Borosilicate glass pipettes (catalog #BF150-110-10, Sutter Instrument) were pulled using a micropipette puller (P-1000, Sutter Instrument). The resistance of the pipettes ranged from 7.0 to 9.5 MΩ (positive pressure condition). FusionRed-positive neurons were recorded as stGtACR1(+) neurons. FusionRed-negative neurons near FusionRed-positive neurons were also recorded as stGtACR1(–) neurons. Neurons that had a resting membrane potential below –55 mV were used for whole-cell recordings. In the current-clamp recording, glass pipette electrodes were filled with a solution containing the following (in mm): 135 K-gluconate, 7 KCl, 10 HEPES, 0.5 EGTA, 4 MgATP, 0.4 Na_2_GTP, and Na2-phosphocreatine, with pH adjusted to 7.2–7.3 with KOH. The liquid junction potentials of the K-gluconate-based internal solution were estimated to be ∼10 mV and were not corrected.

The recording was conducted with a MultiClamp 700B amplifier (Molecular Devices), filtered with a 10 kHz Butterworth filter. The data were sampled at 20 kHz (PCI-MIO-16XE-10, National Instruments) and collected with WinWCP software (University of Strathclyde).

For optogenetic inactivation, the light from a fiber-coupled laser (OBIS LS 594nm, Coherent) was delivered through a fiber patch cable (400–2200 nm, diameter 400 μm, NA 0.39; catalog #M28LO1, Thorlabs) and dilated with a matched achromatic pair lens (f1 = 30.0 mm, f2 = 100.0 mm, 400–700 nm; catalog #MAP1030100-A, Thorlabs). To adjust the laser intensity, an absorptive ND filter (optical density: 1.0; catalog #NE10B, Thorlabs) was placed after the lens. We set the laser intensity to 12.7 mW and 0.24 mW. The laser was illuminated 50° oblique to the surface of the bath. To determine whether the light illumination suppressed the activity of the recorded neurons, the light was illuminated for 1 s during 2 s current injection from the patch pipette to the recorded neuron. To normalize the spike rate of each neuron, the spike rate during photostimulation (1 s) was divided by the spike rate for 0.2 s immediately before each photostimulation onset.

The radius of the laser beam at the slice surface was 0.73 mm, which was similar to the estimated radius of the effective *in vivo* illumination area, as described above. In the *in vivo* optogenetic inactivation with or without imaging, the laser intensity was assumed to have attenuated to ∼10% in L5a (at a depth of ∼0.5 mm from the brain surface) because of scattering and absorption (Yizhar et al., 2011). Thus, the intensity of the laser used in the optogenetic inactivation of IT neurons in the task-performing mice was estimated to be 1.2 mW in L5a, which would be the most effective layer for activating stGtACR1 on L5a IT neurons. In the *in vitro* experiment in which the recorded neurons were located near the slice surface, even 0.24 mW laser illumination strongly inhibited the activity of stGtACR1-expressing IT neurons in L5a, whereas 12.7 mW laser illumination failed to inhibit the activity of L5a neurons that did not express stGtACR1 (see Fig. 6*G–L*). Given the results of the experiment on *in vivo* simultaneous photoinactivation and calcium imaging, we consider that stGtACR1-expressing IT neurons in the behaving mice were effectively and specifically suppressed by the light illumination.

### Histology

Mice were deeply anesthetized with an intraperitoneal injection of ketamine and xylazine and transcardially perfused with PBS, followed by 4% paraformaldehyde (09154-85, Nacalai Tesque). Their brains were removed and submerged in 4% paraformaldehyde for postfixation overnight. Brains were coronally sectioned into 100-µm-thick slices using a Leica VT1000 S Microtome (Leica Microsystems). Fluorescence Nissl staining was performed with NeuroTrace Green Fluorescent Nissl Stain (1:200; catalog #N21480, Thermo Fisher Scientific) or NeuroTrace Deep-Red Fluorescent Nissl Stain (1:200; catalog #N21483, Thermo Fisher Scientific). Epifluorescence images were acquired using a fluorescence microscope (BX53F, Olympus) equipped with a 4× or 10× objective lens and fluorescence filter sets appropriate for GFP, RFP, Texas Red, and Cy5. Software based on the Allen Mouse Brain Common Coordinate Framework was used to identify the position of the Neuropixels 1.0 probe tract in the brain and select the M1 L5 units (Shamash et al., 2018).

### Extraction of ROIs and their activities

The two-photon imaging time series data were motion corrected using the imaging data (using the green channel showing the cell structure imaged from the constitutive expression of GFP), as previously described (Masamizu et al., 2014; Tanaka et al., 2018). The shifts calculated for the correction were applied to the red channel (jRGECO1a), resulting in motion correction analysis independent of neural activity in the red channel.

ROIs and time series calcium traces (Δ*F*/*F*) were extracted using the CaImAn algorithm (Giovannucci et al., 2019) in MATLAB (MathWorks). Extracted calcium traces were deconvolved using the constrained AR-2 OASIS algorithm (Friedrich et al., 2017), resulting in deconvolved calcium traces. To define pursued neurons, ROIs in the same field of view over consecutive days were visually aligned according to their spatial relationship with centroid positions (Masamizu et al., 2014).

### Body part tracking

The DeepLabCut toolbox was used to extract movements of the lever, right forepaw, left forepaw, jaw, and tongue from movies acquired during the motor task (Mathis et al., 2018). For right- and left-forepaw movements, we tracked the middle digit of the right and left forepaws, respectively. The forepaw movements were considered as forelimb movements. Learning with small training sets (20–50 frames per movie) was sufficient to track movements in almost all video frames. In some frames where mice extended their forelimbs to the spout, their forepaws occluded the jaw and tongue, and the likelihood of jaw and tongue tracking was low, and such frames were therefore omitted from the following analyses. Additionally, in some frames, the forepaws were difficult to track (likelihood <0.99), but these frames were rare (0.32 ± 0.39% of all frames for the right forepaw, 0.18 ± 0.26% for the left forepaw, *n* = 24 sessions; mean ± SD). In sporadic single frames with vague forepaw positions, the forepaw positions were interpolated from the frames before and after the frame in question. Consecutive frames with ambiguous forepaw positions were omitted from the following analyses. The total percentage of omitted frames was 0.81 ± 0.81% (*n* = 24 sessions; mean ± SD). The component proportional to the lever movement was subtracted from the right-forepaw movement, allowing us to extract the lever movement-independent right-forepaw movement, RF_lever(–)_. The constant gain for the subtraction was determined for each session as that minimizing the deviation of the subtracted right-forepaw movement.

### Behavioral and neuronal variables

For a time point t, we used x as the lever position (one axis) and $\tilde{x}$ as all the other behavioral variables, namely, lever velocity (two directions), position (two axes) and velocity (four directions) of right and left forepaws, position (one axis) and velocity (two directions) of jaw movement, licking (0 or 1), and reward information (0 or 1). Thus, we had 20 behavioral variables for a time point. For neuronal variables, we binarized the deconvolved calcium traces according to a threshold of 0.005 per imaging frame (binarized activity) and used $r_{i}$ to represent the binarized activity (0 or 1) of neuron i at a time point t.

### Encoding model

We first created a spike encoding model, $P\left(r_{i}\text{|}x,\tilde{x}\right)$ using an elastic net generalized linear model (GLM) with a binomial distribution. To capture the temporal relationships between behavioral variables and the binarized activity, we convolved all variables with 21 evenly spaced Gaussian basis functions (centers of functions set to ± 3.0, 2.7, 2.4, 2.1, 1.8, 1.5, 1.2, 0.9, 0.6, 0.3, and 0 s; scale variable to 0.2 s; the half-width at half-height of these functions was 0.52 s; Runyan et al., 2017). If an impulse was expanded with these functions, neighboring signals would have a correlation of at least 0.53, and this moderate correlation allowed stable fitting. The Bernoulli elastic net GLM fitting was performed using the lassoglm function in MATLAB. The elastic net regularization was based on a combination of L1- and L2-type regularization with a parameter α, such that alpha = 0 corresponds to pure L2 regularization (equivalent to ridge regularization) and alpha = 1 to pure L1 regularization [equivalent to LASSO (least absolute shrinkage and selection operator)]. We used alpha = 0.2 to avoid variable degeneracies while selecting useful variables from potentially correlated ones. Shrinkage parameters were fixed as lambda = 0.0001 for all neurons in all conditions. This value was determined by a preliminary search of parameters with 10-fold cross-validation to maximize obtaining good fitting results.

When an encoding model of a binarized activity train was made, the training frames and test frames from all imaging frames in each session were sampled. The ratio of the number of positive frames (the binarized activity = 1) to the number of negative frames (the binarized activity = 0) was 0.0061 ± 0.0051 (*n* = 3180 neurons, mean ± SD). To avoid an excessive imbalance between the numbers of positive and negative training frames, we chose nine-tenths of the positive frames in order and randomly selected 1% of the negative frames for training (Hoens and Chawla, 2013). After training, we estimated the probability of binarized activity in the residual one-tenth of positive frames and all negative frames. To minimize data leakage between training and test frames, we did not use frames 2 s before or after the training frames used for the test. We repeated this procedure 10 times to test all positive frames, and median values were assigned for the negative frames that were estimated multiple times. Then, we concatenated all data frames to obtain a single train of estimated probability for the target binarized activity, and all performances described below were cross-validated. For a null model (Runyan et al., 2017), we fitted each binarized activity train with a single constant parameter, otherwise in the same way as above.

### Performance and consistency of the encoding models

We use the term “full model” to refer to the encoding model constructed with all 20 behavioral variables. First, we compared the cross-validated likelihood of full models with that of the corresponding null model. The means of the cross-validated likelihood of full and null models per frame were 0.967 ± 0.024 and 0.960 ± 0.027, respectively (*n* = 3180, mean ± SD). In 2790 of these neurons, the cross-validated likelihood was higher in the full model than in the null model. We defined 390 neurons with full models that did not outperform the null model as “elusive neurons.” Excluding these elusive neurons, we calculated the square of the correlation coefficient between a trial-averaged recorded trace and the corresponding estimate within the task-related period (from 1 s before to 3 s after the successful lever-pull initiation) as the prediction accuracy (cross-validated *R*^2^) of the task-related activity.

The 20 variables were classified into the six categories (lever, right forepaw, left forepaw, jaw movement, licking and reward information). To assess the contribution of the set of behavioral variables in each category, we made two types of models. The contribution of a category of variables to the statistical model was maximized when only that category was used, so we made a single category model to assess the maximum contribution of these variables. In contrast, the difference in performance between the full model and models without a single category of variables, which we refer to as the “encoding unique contribution,” shows the minimum contributions of these variables. We calculated the encoding unique contribution for every category by subtracting the performance of the model without the single category from the performance of the full encoding model. When the calculated result for a neuron was below zero, the encoding unique contribution of the category in that neuron was set to zero. We excluded the elusive neurons from these analyses.

The normalized rank of neuronal encoding performance of neuron *i* (*i* = 1, 2, …, *N*) in a session was calculated as (*N* − *R_i_* + 1)/*N*, where *N* is the number of extracted neurons in that session, and *R_i_* is the rank of neuron *i*. Hence, the top performing neuron in a session had a normalized rank of 1, and conversely, the worst performing neuron in a session had a normalized rank of 1/*N*. In this normalization, the performance of an elusive neuron was set to 0, and thus its normalized rank was forcedly set as the smallest value in that session. The number of observed active neurons in LS13 in each animal was 58–229 (*n* = 6 mice), and therefore the number of neurons with a normalized rank of 1–0.8 in LS13 was 12–46. If a pursued neuron had a normalized rank of 1–0.8 in LS13 for each animal, it was classified as a top 20% neuron. The number of top 20% neurons in each animal was 8–22 (6 mice) and their total number was 81. The proportion of top 20% neurons to all neurons with a normalized rank of 1–0.8 in LS13 was 0.24–0.65. Then, we calculated the change in the normalized rank of these top 20% neurons in LS14, TS1, and TS2. Assuming that the distribution of the ranks of nonpursued neurons was similar between different sessions, we consider that the estimation of the ranks of the pursued neurons across sessions is valid, although the nonpursued neurons were not consistently the same across the four sessions.

We analyzed the kernel of the model to assess how each behavioral variable contributed consistently to the encoding model. For each variable, we had weights for various Gaussian basis functions, and from these weights and functions we reconstructed the temporal dependency of the model on the behavioral variable, which we refer to as a kernel. As we made an encoding model for each training spike train in the 10-fold cross-validation, we had 10 spike train models for a single session. Therefore, the median value of these 10 kernels was first calculated, then the between-session correlation coefficients were calculated for every variable of the kernel and averaged across pursued neurons (the top 20% neurons or other neurons).

### Decoding model

In the following, we describe the method for decoding x, while marginalizing the other behavior variables $\tilde{x}$. We estimated x as the average of all possible values as follows:

(1) $$\hat{x}=\sum xxP\left(x\text{|}\underline{r}\right),$$

where $\hat{x}$ denotes an estimation of x. In this equation, we want to know $P\left(x\text{|}\underline{r}\right)$, which means the probability of x with the observation of population activity $\underline{r}$. From herein, we denote the observed binarized activity of neuron i as ${\underline{r}}_{i}$ and the observed population binarized activity as $\underline{r}$ for clarity. This probability is the marginal of the full description of the relationship between behavioral variables and population activity as follows:

(2) $$P\left(x\text{|}\underline{r}\right)=\underset{\tilde{x}}{\sum }P\left(x,\tilde{x}\text{|}\underline{r}\right).$$

Bayes's theorem leads to the following:

(3) $$P\left(x,\tilde{x}\text{|}\underline{r}\right)=\frac{P\left(\underline{r}\text{|}x,\tilde{x}\right)P\left(x,\tilde{x}\right)}{\sum^{}P\left(\underline{r}\text{|}x,\tilde{x}\right)}.$$

Hence, we get the following:

(4) $$P\left(x\text{|}\underline{r}\right)=\underset{\tilde{x}}{\sum }\frac{P\left(\underline{r}\text{|}x,\tilde{x}\right)P\left(x,\tilde{x}\right)}{\sum^{}P\left(\underline{r}\text{|}x,\tilde{x}\right)}$$

and

(5) $$\hat{x}=\sum x,\tilde{x}x\frac{P\left(\underline{r}\text{|}x,\tilde{x}\right)P\left(x,\tilde{x}\right)}{\sum^{}P\left(\underline{r}\text{|}x,\tilde{x}\right)}.$$

$P\left(\underline{r}\text{|}x,\tilde{x}\right)$ is the likelihood of observed population activity based on our encoding models and is expressed using the encoding results, $P\left(r_{i}\text{|}x,\tilde{x}\right)$, as follows:

(6) $$P\left(\underline{r}\text{|}x,\tilde{x}\right)=\prod in1-\left|{\underline{r}}_{i}-P\left(r_{i}\text{|}x,\tilde{x}\right)\right|.$$

All realizations of the combination of $x,\tilde{x}$ that we have are only within a time series of sessions. Therefore, if we permit $P\left(x,\tilde{x}\right)$ to have equal probability, k, for a value realized in a time series and zero for a value not realized in each session, following Runyan et al., 2017, $\hat{x}$ can be best estimated in this framework as follows:

(7) $${\hat{x}}_{t}=k\sum sx_{s}\frac{P\left({\underline{r}}_{t}\text{|}x_{s},{\tilde{x}}_{s}\right)}{\sum_{s}P\left({\underline{r}}_{t}\text{|}x_{s},{\tilde{x}}_{s}\right)},$$

where

(8) $$P\left({\underline{r}}_{t}\text{|}x_{s},{\tilde{x}}_{s}\right)=\prod in1-\left|{\underline{r}}_{i,t}-P\left(r_{i,s}\text{|}x_{s},{\tilde{x}}_{s}\right)\right|.$$

We also incorporated neuronal dynamics into our model by using multiple frames of populational binarized activities; that is, we substituted ${\underline{r}}_{t}$ with a concatenated vector, $\left[{\underline{r}}_{t-6},{\underline{r}}_{t-4},{\underline{r}}_{t-2},{\underline{r}}_{t},{\underline{r}}_{t+2},{\underline{r}}_{t+4},{\underline{r}}_{t+6}\right]$, for the actual estimation (here, ${\underline{r}}_{t-\tau }$ represents the population binarized activity at τ frames before frame t). We estimated $\hat{x}$ accordingly, and the summation in Equation 7 was performed at all time points excluding those 3.3 s before and after the time points in question to eliminate data leakage.

### Decoding contribution

The decoding contribution of a specific neuronal population was tested as the decoding unique contribution of each population. In each session for each animal, we calculated the decoding performances of the lever trajectories from all pursued neurons and from all pursued neurons except for the top 20% neurons. For each session, the difference between these two decoding performances (after animal averaging) was calculated as the decoding unique contribution of the top 20% neurons. To determine whether their contribution was higher than that of other possible populations comprised of nontop 20% neurons in each session, we randomly chose the same number of neurons from nontop 20% pursued neurons as for the top 20% neurons and calculated the animal-averaged Δ*R*^2^ of these neurons in each session. We calculated the Δ*R*^2^ of these random control populations 100 times and estimated the distributions of Δ*R*^2^ for nontop 20% pursued neurons with histograms.

### Experimental design and statistical analysis

All statistical analyses were performed using MATLAB (versions R2021b, R2022a, R2022b). Sample sizes were chosen according to previous studies. Male mice were used in all experiments except for an *in vitro* acute slice experiment. Unless otherwise noted, data are summarized as the mean ± SEM. Parametric tests were primarily used for statistical tests, but nonparametric tests were also used when the distribution of data obviously differed from a normal distribution (see Fig. 4*G*,*H*). All *p* values were calculated with a two-tailed distribution and were corrected for multiple comparisons when necessary. Numerical methods (bootstrap range and shuffling) were also used when we could not make any assumptions about the data distribution (see Figs. 4*A*, 5*A*,*B,E–G*). For the statistical analyses visualized in the figures, detailed statistical designs are presented in the Figure legends.

## Results

### Learned motor skill is retained after 6 d of nontraining

**Figure 1.**
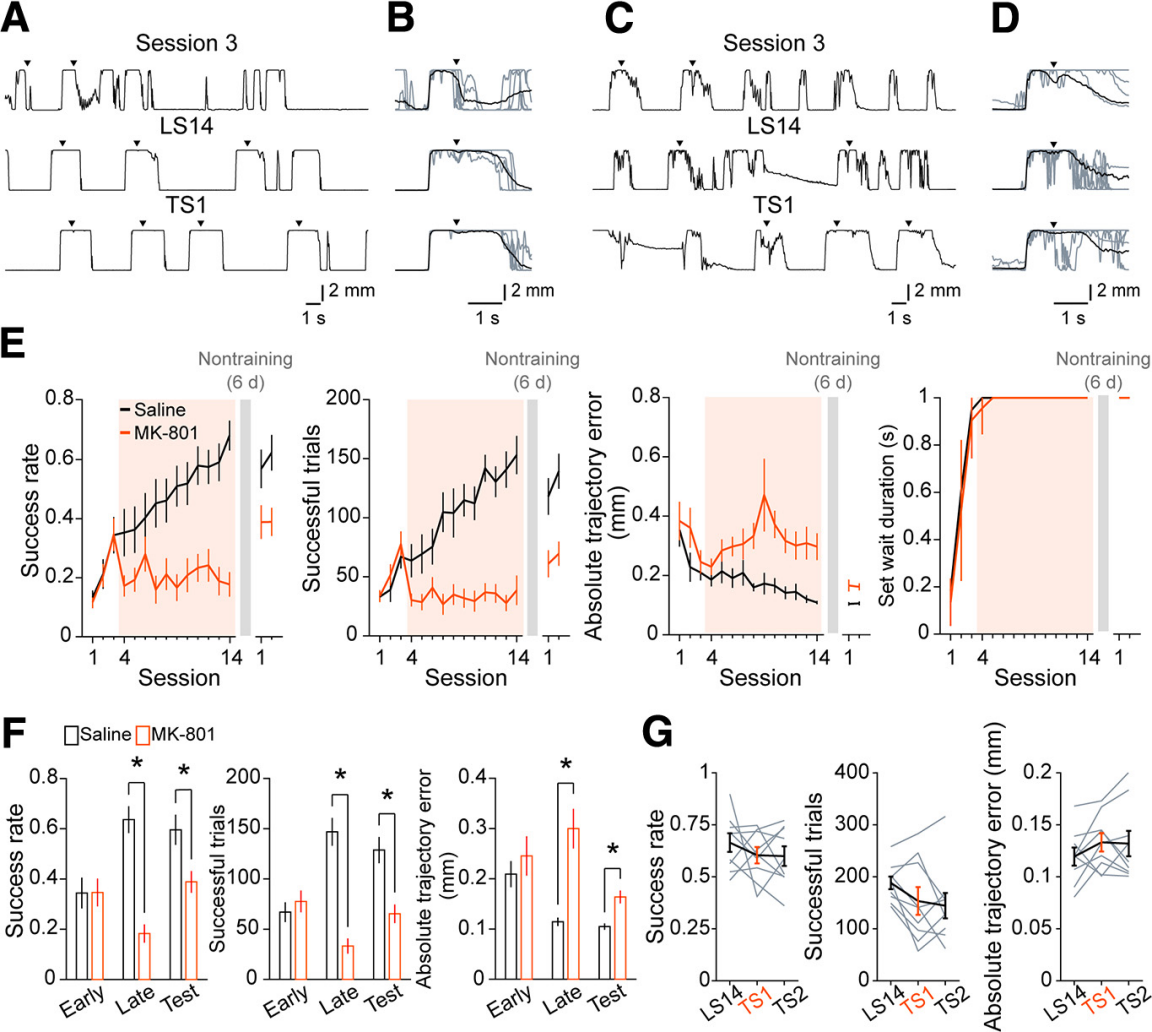
Learning of a self-initiated lever-pull task and performance after the nontraining days with or without NMDA antagonist treatment. ***A***, Lever trajectories in the third learning session (session 3), LS14, and TS1 from a representative mouse in the saline-solution-administered control group. ***B***, Lever trajectories of five consecutive trials (gray) and the mean lever trajectories in successful trials (black) in each session shown in ***A***. Arrowheads indicate the reward timings. ***C***. Lever trajectories in session 3, LS14, and TS1 from a representative mouse in the MK-801-solution-administered group. ***D***, Lever trajectories of five consecutive trials (gray) and the mean lever trajectories in successful trials (black) in each session shown in ***C***. ***E***, Task performance in the learning and test sessions with NMDAR antagonist treatment between the middle and late learning sessions (the fourth to 14th learning sessions). From left to right, success rate, number of successful trials, mean absolute trajectory errors, and minimum set wait duration are shown. Trajectory errors were calculated in successful trials for 0.6 s from the lever-pull initiation from the reference expert lever trajectory which was defined as the mean lever trajectory in successful trials in LS13 and LS14 from mice in the saline solution-administered control group. *n* = 11 mice in the saline-solution-administered control group, *n* = 13 mice in the MK-801-solution-administered group. ***F***, A summary of task performance in the early (session 3) and late learning sessions (LS13 and LS14) and test sessions (TS1 and TS2). Left, Mean success rate. Middle, Mean number of successful trials. Right, Mean absolute trajectory errors from the expert lever trajectory in successful trials at 0.6 s from the lever-pull initiation, *p < 0.05 by Welch's *t* test with Bonferroni correction, *n* = 11 mice in the saline-solution-administered control group, *n* = 13 mice in the MK-801-solution-administered group. ***G***, Task performance with NMDAR antagonist treatment in TS1. Left, Success rate, *p* > 0.05 across sessions, paired *t* test with Bonferroni correction, *n* = 9 mice. Middle, Number of successful trials, *p* > 0.05 across sessions, paired *t* test with Bonferroni correction, *n* = 9 mice. Right, Mean absolute trajectory errors in successful trials, *p* > 0.05 across sessions, paired *t* test with Bonferroni correction, *n* = 9 mice.

We trained head-fixed mice to perform the self-initiated lever-pull task (Tanaka et al., 2018). After 14 learning sessions, we left a 6 d nontraining period (the nontraining days) over which the trained animals were not exposed to the task. Then, the mice were re-exposed for additional sessions (test sessions). We previously demonstrated that a subset of L5a neurons in the CFA increase the motor representation from middle to late learning sessions (Masamizu et al., 2014), with this being related to synaptic plasticity. Therefore, we examined whether the NMDAR, which is a key component of synaptic plasticity in motor learning (Hasan et al., 2013; Albarran et al., 2021), contributes to improvement in task performance after the fourth learning session. We intraperitoneally administered the NMDAR antagonist MK-801 (0.25 mg/kg) to a group of mice in the fourth to 14th learning sessions. In another group that served as the control, saline was administered in the same manner. In the control mice, the lever-pull trajectory on successful trials became precise and reproducible over 14 learning sessions (Fig. 1*A*,*B*; Masamizu et al., 2014; Tanaka et al., 2018). Consistent with this, the task performance improved, specifically the success rate (the ratio of the number of successful trials to the total number of lever-pull trials), and the number of successful trials gradually increased. The absolute trajectory error from the expert lever trajectory decreased, indicating that lever-pull movements became stereotyped, and the set wait duration reached a plateau of 1 s by the fifth learning session at the latest (Fig. 1*E*). The task performance was similar not only in the two late learning sessions (LS13 and LS14), but also in the test sessions (TS1 and TS2). This indicates that the task performance attained in the late learning sessions was retained after the nontraining days. By contrast, the MK-801 administration inhibited improvement in the success rate and number of successful trials, and inhibited the decrease in the error of the lever-pull trajectory (Fig. 1*C–E*). The task performance measures in the late learning sessions differed significantly between MK-801-administered and control mice (Fig. 1*F*; Welch's *t* test with Bonferroni correction, the mean success rate, *t*_(18.2)_ = 7.05, adjusted *p* = 3.9 × 10^−6^; the mean number of successful trials, *t*_(15.7)_ = 7.23, adjusted *p* = 6.8 × 10^−3^; the mean absolute trajectory errors from the expert lever trajectory in successful trials at 0.6 s from the lever-pull initiation, *t*_(11.9)_ = −4.43, adjusted *p* = 2.5 × 10^−3^). The differences in performance were still noticeable in test sessions in which both groups performed the lever-pull task without MK-801 (Fig. 1*F*; Welch's *t* test with Bonferroni correction, the mean success rate, *t*_(18.8)_ = 2.80, adjusted *p* = 0.03; the mean number of successful trials, *t*_(18.6)_ = 3.97, adjusted *p* = 2.6 × 10^−3^; the mean absolute trajectory errors from the expert lever trajectory in successful trials at 0.6 s from the lever-pull initiation, *t*_(17.0)_ = −4.13, adjusted *p* = 2.1 × 10^−3^). To evaluate the direct effect of MK-801 on the skilled lever-pull movement, we injected MK-801 into the trained mice only in TS1, where there was no statistically significant effect on the task performance (Fig. 1*G*; paired *t* test with Bonferroni correction, the mean success rate, LS14 vs TS1, *t*_(8)_ = 0.91, adjusted *p* = 0.8; LS14 vs TS2, *t*_(8)_ = 0.95, adjusted *p* = 0.7; TS1 vs TS2, *t*_(8)_ = 0.096, adjusted *p* = 1; the number of successful trials, LS14 vs TS1, *t*_(8)_ = 1.87, adjusted *p* = 0.20; LS14 vs TS2, *t*_(8)_ = 2.24, adjusted *p* = 0.12; TS1 vs TS2, *t*_(8)_ = 0.41, adjusted *p* = 1; the mean absolute trajectory errors in successful trials, LS14 vs TS1, *t*_(8)_ = −1.55, adjusted *p* = 0.32; LS14 vs TS2, *t*_(8)_ = −1.19, adjusted *p* = 0.54; TS1 vs TS2, *t*_(8)_ = 0.15, adjusted *p* = 1). Although it was unclear which brain areas were affected by the MK-801 administration, these results suggest that NMDAR-dependent processes are required for acquisition of the skilled lever-pull movement during middle to late learning sessions, rather than for its execution after learning.

### Learned motor behavior was dependent on CFA

**Figure 2.**
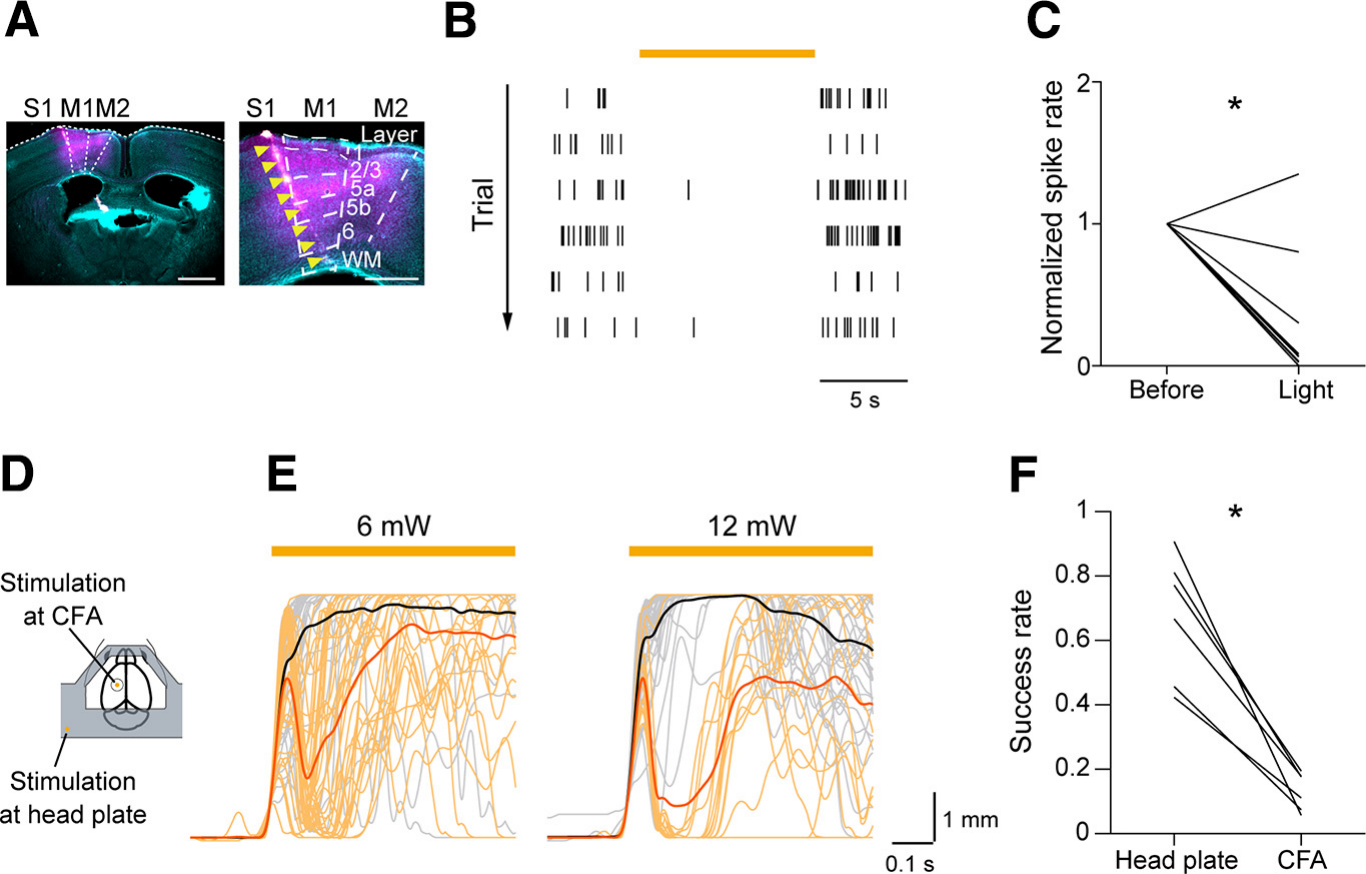
Inhibition of skilled lever pulls caused by CFA optogenetic manipulations in the test sessions. ***A***, Left, Histological image of the Neuropixels 1.0 probe tract in a coronal section including the CFA that was 0.2 mm anterior to the bregma. Cyan indicates neurons labeled with NeuroTrace. Magenta indicates stGtACR1-FusionRed-expressing neurons. The Neuropixels 1.0 probe was labeled with CM-Dil (indicated by yellow arrowheads). Scale bar, 1 mm. Right, Magnification of the image on the left. Scale bar, 0.5 mm. WM, white matter. ***B***, Raster plots of spikes from a representative M1 L5 unit in six light illumination trials at 12 mW. The orange bar indicates the light illumination. ***C***, A summary of normalized spike rates of M1 L5 units before and during light illumination, *p = 6.4 × 10^−4^ by paired *t* test, *n* = 10 units. ***D***, Schematic image of the light illumination sites on the head plate and CFA. ***E***, Left, Lever traces in optogenetic stimulation trials at 6 mW. Lever traces in the head plate illumination trials (*n* = 37 trials, black) and in the CFA illumination trials (*n* = 34 trials, orange) from a representative mouse. Right, Lever traces in optogenetic stimulation trials at 12 mW. Lever traces in the head plate illumination trials (*n* = 40 trials, black) and in the CFA illumination trials (*n* = 16 trials, orange) from a representative mouse, the same mouse as on the left. Mean lever trajectories in each type of trial are shown as thick lines. The orange bar indicates light illumination. ***F***, Summary of the success rate in the head plate illumination trials and the CFA illumination trials at 6 mW, *p = 1.0 × 10^−3^ by paired *t* test, *n* = 6 sessions from 3 mice.

As we previously found that skilled lever-pull movements require M1 in the late stage of learning (Hira et al., 2013; Tanaka et al., 2018; Terada et al., 2022), it seemed sensible to test whether skilled lever-pull movements depend on M1 after the nontraining days. Therefore, we optogenetically silenced the activity of excitatory neurons in the CFA in the test sessions. To inactivate CFA, we injected AAV encoding soma-targeted *Guillardia theta* anion-conducting channelrhodopsin (*AAV-stGtACR1-FusionRed*; Mahn et al., 2018) into the left CFA before the learning sessions started. We confirmed that continuous 594 nm laser illumination on the CFA reduced the spike rates of deep-layer neurons (Fig. 2*A–C*; paired *t* test, *t*_(9)_ = 5.10, *p* = 6.4 × 10^−4^; Fig. 2*C*).

To evaluate the contribution of M1 during lever-pull movements, we established a closed-loop stimulation protocol during the lever-pull task. When the lever was pulled beyond 1 mm from its natural position, the laser was irradiated to either the left CFA (25% of trials) or the head plate (25% of trials, for the control experiment) to evaluate the effect of silencing CFA on the lever-pull behavior (Fig. 2*D*; Hwang et al., 2019; Terada et al., 2022). The laser was not applied in the rest of the trials (50% of trials). When the laser was illuminated on the CFA in the test sessions, the lever position was rapidly returned to the natural position and was then moved to the pull direction again (Fig. 2*E*). The lever was repulled more frequently with the laser illumination set at 6 mW than when it was set at 12 mW, suggesting that the inhibitory effect on lever pulling was stronger with 12 mW illumination than with 6 mW (Fig. 2*E*). Because the perturbation interrupted continuous pulls, the success rate in trials with laser illumination on the CFA was much lower than that in trials with laser illumination on the head plate (Fig. 2*F*; paired *t* test, *t*_(5)_ = 6.87, *p* = 1.0 × 10^−3^). Thus, skilled lever-pull movements after the nontraining days required CFA activity.

### Two-photon imaging of L5a intratelencephalic neurons in CFA

**Figure 3.**
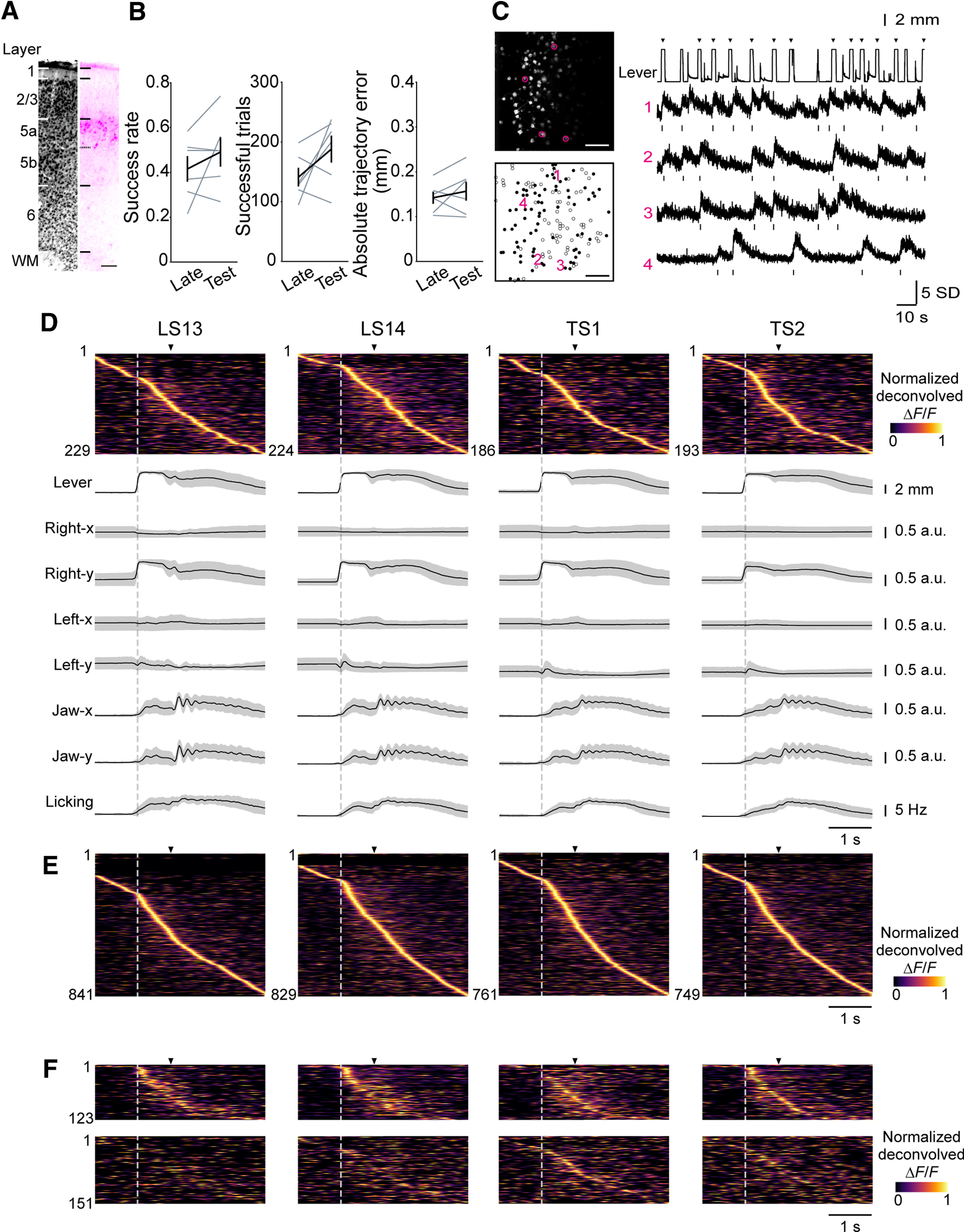
Longitudinal two-photon calcium imaging of CFA L5 IT neurons. ***A***, Histological image of jRGECO1a expressed in a representative *Tlx3-Cre* mouse. Left, Neurons labeled with NeuroTrace. Right, Neurons expressing jRGECO1a. Scale bar, 100 µm. WM, white matter. ***B***, A summary of task performance in the late learning sessions (LS13 and LS14) and test sessions (TS1 and TS2). Left, Mean success rate, *p* = 0.27 by paired *t* test. Middle, Mean number of successful trials, *p* = 0.14 by paired *t* test. Right, Mean absolute trajectory errors in successful trials of the expert lever trajectory at 0.6 s from the lever-pull initiation, *p* = 0.42 by paired *t* test, *n* = 6 mice. ***C***, Left, Frame-averaged two-photon image of L5a IT neurons expressing jRGECO1a in the CFA from a representative mouse (top). ROIs extracted by the CaImAn algorithm. Left, Neurons tracked throughout the imaging sessions (pursued neurons) are shown as black circles, and other neurons are shown as white circles (bottom). Scale bar, 100 µm. Right, Representative lever trajectories and Δ*F/F* traces of neurons 1–4 in the left images. Frames with the deconvolved calcium activity are shown in raster plots below each trace. Arrowheads indicate the reward timings. ***D***, Trial-averaged deconvolved calcium traces (normalized) of CFA L5a IT neurons and movements of body parts (lever, right forelimb, left forelimb, jaw, and licking) from a representative mouse aligned to lever-pull initiation of successful trials (gray dotted line). Arrowheads indicate reward timings. ***E***, Trial-averaged deconvolved calcium traces (normalized) of CFA L5a IT neurons pooled from six mice and aligned to the lever-pull initiation of successful trials. ***F***, Trial-averaged deconvolved calcium traces (normalized) of CFA L5a IT pursued neurons aligned to the lever-pull initiation of successful trials (*n* = 274 cells from 6 mice). Top, The mean sorted deconvolved calcium traces (normalized) of the fraction of pursued neurons whose mean correlation of trial-averaged deconvolved calcium traces (normalized) across imaging sessions was higher than that of the 95th percentile of random shuffling (the 95th percentile of the correlation coefficient was 0.136, *n* = 123 neurons). Bottom, The mean sorted deconvolved calcium traces (normalized) of the remaining fraction of pursued neurons (*n* = 151 neurons). The order of neurons is the same across sessions.

**Figure 4.**
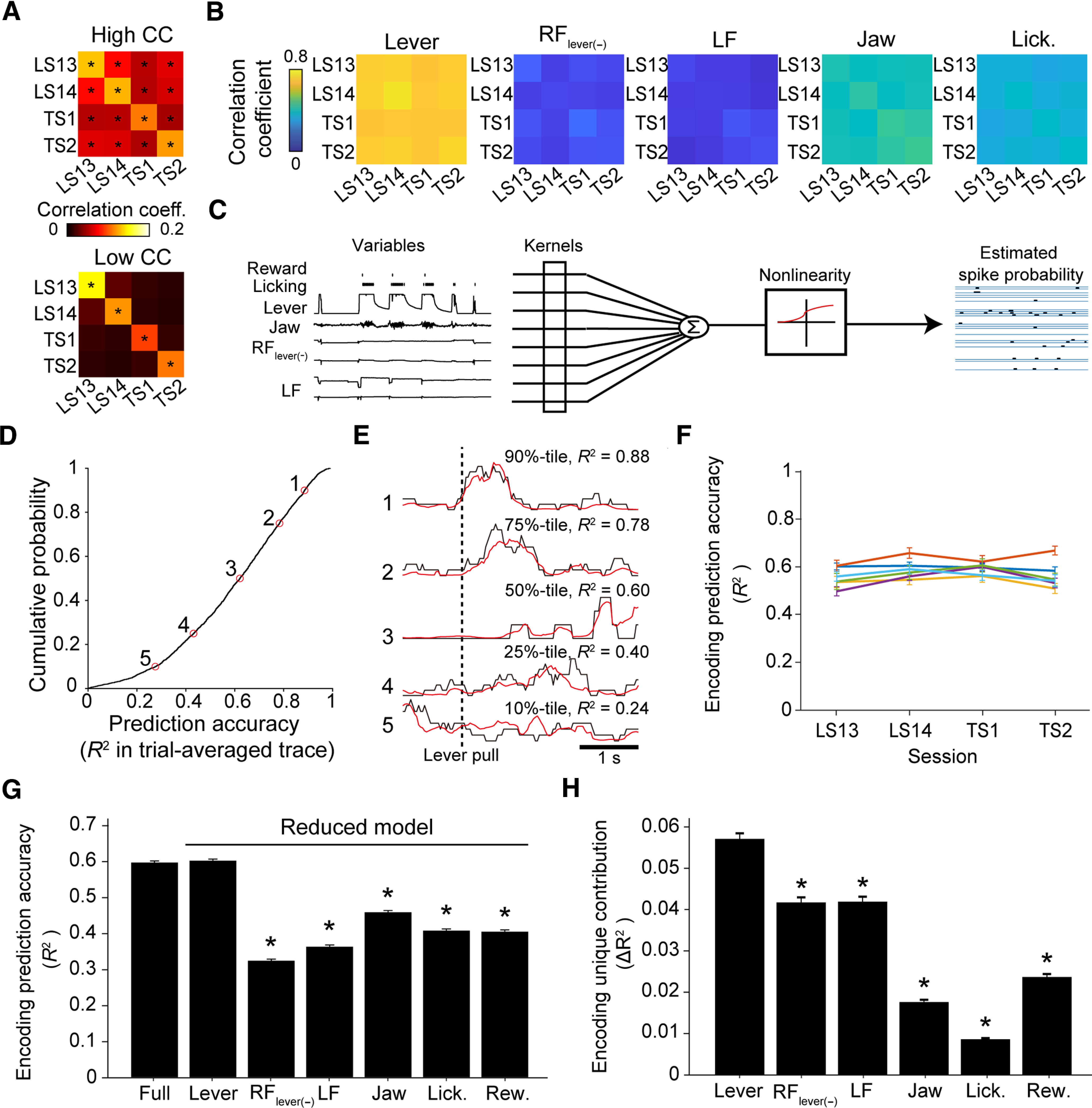
Lever movement was well encoded in CFA L5a IT neurons during the motor task. ***A***, Correlation coefficients of the activities of pursued neurons. Top, The average correlation coefficient of the neurons shown in Figure 3*F* (top; *n* = 123 neurons). Bottom, The correlation coefficients of neurons shown in Figure 3*F* (bottom; *n* = 151 neurons). The correlation coefficients were averaged across 1000 randomly selected trial-by-trial correlations from before 1 s to after 3 s relative to the lever-pull initiation (*p < 0.05 vs time-shifted control). ***B***, Correlation coefficients of task variables averaged across 1000 randomly selected trial-by-trial correlations (1000 instances from each of six mice). ***C***, Schemes of the encoding model of L5a IT neurons during the lever-pull task. ***D***, Cumulative probability of the accuracy of the mean binarized activity aligned with lever-pull initiation that was predicted by the encoding models using all variables. Only neurons that were reconstructed by the encoding models with higher prediction accuracy (cross-validated log likelihood per frame; see above, Materials and Methods) than the null model are plotted (*n* = 2790 neurons). ***E***, Mean traces across trials for deconvolved calcium traces (binarized, black), and binarized activity (red) for five representative neurons. The same numerical figures in ***D*** and ***E*** indicate data from the same neuron. ***F***, The variance in the encoding prediction accuracy across animals and days. Different colored lines indicate different mice. Although statistical significance was found in 68/276 possible comparisons (between 6 mice and 4 d; *p* < 0.05, *t* tests with Bonferroni correction), the absolute differences were small overall [0.14 at most, 0.046 ± 0.034 (mean ± SD), *n* = 276 comparisons]. ***G***, The encoding prediction accuracy of the full model (Full). The encoding prediction accuracies of the models with variables in a single category are also shown (Lever, prediction accuracy for the model with only 3 lever-related variables; RF_lever(-)_ for the model with only 6 RF-related variables; LF for the model with only 6 LF-related variables; Jaw for the model with only 3 jaw-related variables; Lick. for the model with only licking timing; Rew. for the model with only reward timing, *n* = 2790 neurons; *p < 0.05 vs full model, signed-rank test with Bonferroni correction). ***H***, Unique encoding contributions (Δ*R*^2^) of single-category variables, calculated by subtracting the prediction accuracy from that of the corresponding full model (e.g., the unique contribution for Lever was calculated by subtracting the model with 17 variables other than the 3 lever-related variables; *n* = 2790 neurons; *p < 0.05 vs Lever, signed-rank test with Bonferroni correction).

To explore the relationships between skilled lever-pull movements and the activity of CFA L5a IT neurons with large projections to the striatum, we labeled these neurons by injecting a Cre-dependent AAV encoding the red calcium indicator jRGECO1a (*AAV-FLEX-jRGECO1a*; Dana et al., 2016) into the left CFA of *Tlx3-Cre* mice (Gerfen et al., 2013). This transfection resulted in strong jRGECO1a expression in L5a (Fig. 3*A*), as previously reported (Gerfen et al., 2018; Park et al., 2022). We trained these mice and conducted two-photon calcium imaging of *Tlx3*+ neurons in CFA at a depth of 371–500 μm from the cortical surface in LS13, LS14, TS1, and TS2. The task performance was not significantly different between the late learning sessions and the test sessions (Fig. 3*B*; paired *t* test, the mean success rate, *t*_(5)_ = −1.24, *p* = 0.27; the mean number of successful trials, *t*_(5)_ = −1.73, *p* = 0.14; the mean absolute trajectory errors from the expert lever trajectory in successful trials at 0.6 s from the lever-pull initiation, *t*_(5)_ = −0.87, *p* = 0.42). We detected changes in Δ*F*/*F* (representing neuronal activity) among a total of 3180 active *Tlx3*+ neurons in the CFA of six *Tlx3-Cre* mice (Fig. 3*C*). A large population of *Tlx3*+ neurons in CFA showed peak trial-averaged deconvolved calcium traces during lever pulling (Fig. 3*D*,*E*), which is consistent with our previous study (Masamizu et al., 2014). Among the imaged active *Tlx3*+ neurons in the CFA, a subset was identified throughout the four imaging sessions (274 neurons from six mice, represented by the black ROIs in Fig. 3*C*), and we refer to these as “pursued neurons.” Of these pursued neurons, 123 showed a significant correlation of trial-averaged deconvolved calcium traces across imaging sessions (with a higher correlation than the 95th percentile of shuffling). These neurons had peak times that were relatively stable across the four sessions and were distributed throughout the lever pulling (Fig. 3*F*). The trial-to-trial correlation of the deconvolved calcium traces of these neurons during the task-related period (from 1 s before to 3 s after successful lever-pull initiation) was high both within and between sessions (Fig. 4*A*). In contrast, the other pursued neurons (151 neurons) had varying trial-averaged peak times and lower trial-to-trial correlation across the four sessions (Figs. 3*F*, 4*A*). These results indicate that a fraction of the CFA L5a IT neurons stably represented skilled lever-pull movements before and after the nontraining days.

### Encoding models captured activity dynamics of CFA L5a IT neurons during the motor task

We previously showed that a subset of L5a neurons in the CFA evolves to represent the lever-pull movement from the middle stage of learning (Masamizu et al., 2014). Therefore, we speculated that the stability of the activity of a subset of CFA L5a IT neurons in the late learning sessions and test sessions might partly reflect the trial-to-trial stability of lever-pull movement. In fact, when the time series of the lever-pull trajectories were aligned to the lever-pull initiations, the trial-to-trial correlation within and between sessions was very high, at ∼0.8 (Fig. 4*B*). In contrast, the trial-to-trial correlation of the lever-irrelevant right forelimb movement (RF_lever(−)_; see above, Materials and Methods”) and left forelimb (LF) movement was very low, even within the same session, being ∼0.1. The orofacial movements (jaw and lick movements) were uninstructed movements but were related to the reward expectation and water consumption accompanying the lever pull. The correlation of the orofacial movements was lower than that of the lever-pull trajectory but higher than that of RF_lever(–)_ and LF movement (Fig. 4*B*). Trial-to-trial variability in the timing of the orofacial movement onsets and the frequency of jaw movement and licking might be larger than that for the lever trajectory. On the basis of these observations, we hypothesized that after learning, the CFA L5a IT neurons would represent the skilled lever-pull movement more strongly and stably than they would represent the uninstructed forelimb movements and orofacial movements.

To test this hypothesis, taking into account the trial-to-trial variability of both lever-related and lever-unrelated movements, and these movements during periods other than successful lever-pull trials, we built generalized linear models to explain the time series of the neuronal activity using 20 behavioral variables classified into the following categories: (1) lever-related variables [one-axis position and two-direction (pull–return) velocities], (2) reward timing, (3) RF_lever(–)_-related variables (two-axis positions and four-direction velocities), (4) LF-related variables (two-axis positions and four-direction velocities), (5) licking timing, and (6) jaw-related variables (one-axis position and two-direction velocities; Fig. 4*C*, full model). For 2790 of the 3180 neurons recorded (from the total of 24 sessions), the full model explained the binarized activity of each neuron better than the null model (see above, Materials and Methods). The ability to predict the binarized activity during the task-related period was estimated as the prediction accuracy (cross-validated *R*^2^) of the trial-averaged activity. These encoding models well captured the time course of trial-averaged binarized activity in each neuron (Fig. 4*D*,*E*), and the encoding performance was similar across days and animals (Fig. 4*F*). These results indicate that our encoding models reliably captured the neuronal activity dynamics of many CFA L5a IT neurons.

Next, we examined the influence of behavioral variables on the modeled activity of each neuron. To estimate the maximum contribution of each category of behavioral variables (such as lever-related variables), we made a separate model for each category. The encoding prediction accuracy of the model with lever-related variables was the highest among the models with a single category of variables and was generally comparable to that of the full model (Fig. 4*G*). In addition, to estimate the minimum contribution of each category, we calculated the encoding unique contribution (Δ*R*^2^), defined as the difference in cross-validated *R*^2^ between the full model and the model without a particular target category of variables (Musall et al., 2019; Terada et al., 2022). Complementing their high contribution to the encoding models, the lever-related variables showed the highest unique contribution of all categories (Fig. 4*H*). These results indicate that in the late learning and test sessions, the neuronal activities of the CFA L5a IT neurons were more influenced by the skilled lever-pull movement than by the other movements.

### The relationship between the learned motor skill and the activity of a subset of L5a IT neurons in CFA remained after the nontraining days

**Figure 5.**
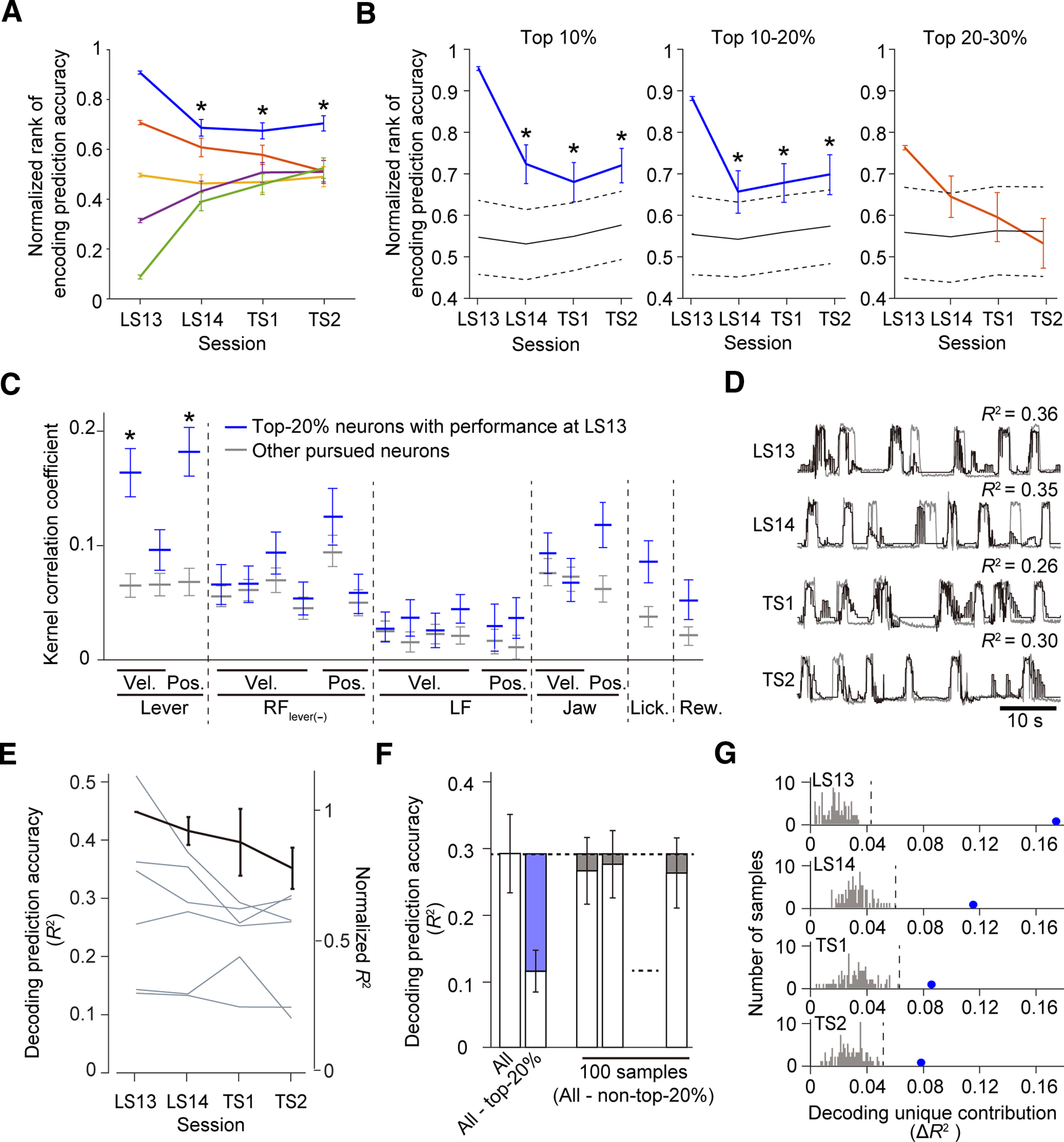
A subset of CFA L5a IT neurons stably encoded the learned motor skill after the nontraining days. ***A***, Normalized rankings of the five groups of pursued neurons in the four imaging sessions. Five groups were determined according to the normalized rankings among the pursued and nonpursued neurons in each imaging field in LS13. The top 20% neurons maintained their rank in the field (blue; *n* = 81 neurons). Other neurons quickly merged to ∼0.5 (orange, neurons with a normalized rank of 0.6–0.8 at LS13, *n* = 49 neurons; yellow, neurons with a normalized rank of 0.4–0.6 at LS13, *n* = 48 neurons; purple, neurons with a normalized rank of 0.2–0.4 at LS13, *n* = 44 neurons; green, neurons with a normalized rank of 0–0.2 at LS13, *n* = 52 neurons). The statistical significance (asterisks) of the top 20% neurons was determined as follows. In each LS14, TS1, and TS2, the average normalized rank was calculated 10,000 times with randomly selected populations of the same size as the top 20% neurons. When the normalized rank exceeded the 97.5th percentile of the distribution in the 10,000 instances, it was taken as being statistically significant. ***B***, Normalized rankings of the top 10% (left), top 10–20% (middle), and top 20–30% (right) neurons in the four imaging sessions. Either a blue or orange line shows the averaged encoding performance of the neurons in question (left, *n* = 41 neurons; middle, 40; right, 27). In each LS14, TS1, and TS2, the average normalized rank was calculated 10,000 times with randomly chosen populations of the same size as the corresponding group of neurons. Black solid lines show the average of 10,000 repetitions for the encoding performance of the randomly chosen neurons. Black dashed lines show 97.5% tile and 2.5% tile of the averaged performance of 10,000 samples. Statistical significance (asterisks) was determined by whether the blue line exceeded the black dashed line. Note that the comparison was based on the mean values but not on the SE bars of the blue line. ***C***. The correlations of the encoding kernels (blue, top 20% neurons, *n* = 81; gray, the other pursued neurons, *n* = 193 neurons; *p < 0.05 by *t* test, between blue and gray, with Bonferroni correction). ***D***, Representative traces of recorded (gray) and predicted (black) lever trajectories (decoded with 33 pursued neurons). ***E***, Daily variations in the decoding prediction accuracy of each mouse are shown by gray lines (each field contained 57, 41, 39, 78, 26, or 33 pursued neurons, scale on the left axis). The black line represents the normalized mean across mice (*n* = 6 mice, scale on the right axis; *p* > 0.14 between days and *p* > 0.17 between mice by one-way ANOVA). ***F***, Schematic of the calculation of decoding unique contributions (Δ*R*^2^) using data from LS13 (6 mice). The blue part of the second column displays Δ*R*^2^ of the top 20% neurons, which was defined as the difference between the decoding performance (*R*^2^) of all pursued neurons (far left column) and that of the pursued neurons other than the top 20% neurons (second column, white part). The first three columns exemplify control samples. The gray parts show Δ*R*^2^ of the subpopulations that were randomly chosen from the nontop 20% neurons that were defined as neurons with a normalized rank of 0–0.8 in LS13. The number of chosen neurons was the same as that for the top 20% neurons. To obtain the distribution of Δ*R*^2^ of subpopulations without the top 20% neurons, the random sampling and calculation were repeated 100 times. ***G***, Histogram of Δ*R*^2^ of the nontop 20% neurons (gray, 100 samples) with Δ*R*^2^ of the top 20% neurons (blue dot, the average across 6 mice) in each session. Each dashed line indicates the highest Δ*R*^2^ of the nontop 20% neurons in each session.

In our previous study (Masamizu et al., 2014), only approximately one-fifth of the top 20% of CFA L5a neurons that strongly possessed information on the lever movement in the early stage of learning were in the top 20% in the late stage of learning. In addition, one-third of CFA L5a neurons evolved to contribute substantially to the ensemble prediction of lever movement in the late stage of learning. Here, we hypothesized that if the motor memory ensemble formed in the late stage of learning is stable, the top 20% of CFA L5a neurons in the late stage of learning would maintain the task-relevant motor information over nontraining days. Therefore, we examined whether CFA L5a neurons with high ranks according to the full model prediction accuracy maintained their high ranks across imaging sessions. The rank was defined according to the encoding prediction accuracy in all neurons, regardless of whether they were pursued or not, and was normalized between 1 (top) and 1/*N* (bottom, *N* means the number of analyzed neurons in the session) in each imaging session. The pursued neurons with the top 20% prediction accuracy in LS13 (top 20% neurons) retained their ranks in subsequent imaging sessions, whereas the ranks of the remaining fraction of pursued neurons varied with a degree of randomness (Fig. 5*A*). Furthermore, when the ranks of pursued neurons that had a normalized rank of 1–0.9 in LS13 (top 10% neurons) and the pursued neurons that had a normalized rank of 0.9–0.8 in LS13 (top 10–20% neurons) were calculated in each session of LS14, TS1, and TS2, they were significantly higher than the averaged rank of randomly chosen neurons in each session (Fig. 5*B*). By contrast, in any of the three sessions, the ranks of the pursued neurons that had a normalized rank of 0.8–0.7 in LS13 (top 20–30% neurons) did not significantly differ from the averaged rank of randomly chosen neurons (Fig. 5*B*). These results suggest that further analyses on the coding characteristics of the top 20% neurons would be reasonable for investigating how motor information is stably maintained in L5a neurons. The contribution of each behavioral variable to the encoding model was expressed by the temporal kernel used to convolute each variable. Therefore, to assess the consistency of the contributions of variables in the pursued neurons, we calculated the between-session correlations of kernel shapes for each behavioral variable. The top 20% neurons maintained the kernel shape of lever-related variables more consistently across imaging sessions than did the other pursued neurons (Fig. 5*C*). These results suggest that highly ranked neurons maintain a more stable relationship between their activity and lever kinematics than do other neurons.

Because our encoding model with behavioral variables well reconstructed the activity of individual neurons, it should be possible to decode a specific behavioral variable from the reconstructed population activity (Runyan et al., 2017). We therefore decoded the lever trajectory from the binarized activities of pursued neurons in each session (Fig. 5*D*) and found that the decoding prediction accuracy of lever trajectories was comparable between the late learning sessions and test sessions (Fig. 5*E*). To estimate the decoding unique contribution (Δ*R*^2^) of the top 20% neurons to the ensemble representation, we subtracted the decoding prediction accuracy calculated for the ensemble without the top 20% neurons from that calculated for all neurons (Fig. 5*F*). We found that in both late learning sessions and test sessions, the estimated decoding unique contribution of the top 20% neurons was higher than that of other equally sized populations (Fig. 5*G*). These results suggest that a fraction of neurons that strongly encoded motor task information during learning retained the acquired representation after the nontraining days.

### CFA L5a IT neurons support the reproducibility of skilled lever-pull movements

**Figure 6.**
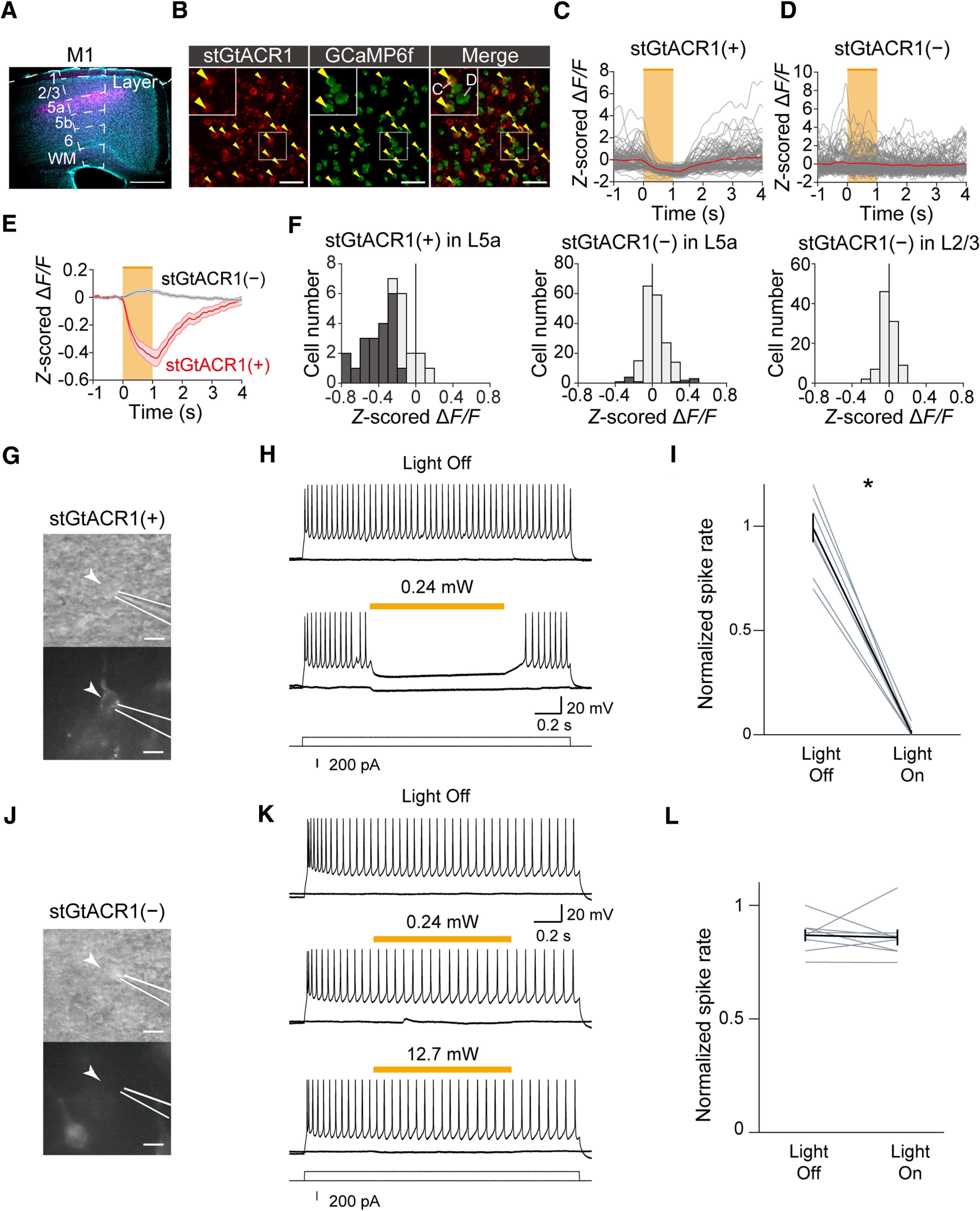
CFA L5a IT neuron-specific optogenetic inactivation. ***A***, A representative coronal image of M1 IT neuron-specific expression of stGtACR1; NeuroTrace (cyan) and stGtACR1-FusionRed (magenta). The image location along the anterior–posterior axis was the same as for the bregma. Scale bar, 0.5 mm. ***B***, Left, Frame-averaged two-photon image of CFA L5a IT neurons expressing stGtACR1-FusionRed. Middle, ROIs of GCaMP6f-expressing neurons that were extracted with the CaImAn algorithm. Right, Merged left and middle images. Scale bar, 50 µm. The yellow arrows indicate the neurons that express both stGtACR1-FusionRed and GCaMP6f [stGtACR1(+) neurons]. Insets, Enlarged view of the area indicated by the smaller white rectangle. ***C***, Representative calcium traces (*z*-scored Δ*F/F*) of stGtACR1(+) neurons shown in ***B*** when the 594 nm light was illuminated (orange). The traces were sorted to the onset of light stimulation. Each gray trace was from each trial, and red traces are the average of all trials. ***D***, Representative calcium traces (*z*-scored Δ*F/F*) of the GCaMP6f-expressing neurons shown in ***B*** that did not express stGtACR1-FusionRed [stGtACR1(–) neurons] when the 594 nm light was illuminated (orange). ***E***, Average response to 594 nm light stimulation of stGtACR1(+) neurons (*n* = 31) and stGtACR1(–) neurons (*n* = 190) in CFA L5a. ***F***, Histogram of the difference between *z*-scored Δ*F/F* during the light stimulation (from 0 to 1 s after the onset of light stimulation) and the baseline (from −1 to 0 s before the onset of light stimulation) in L5a stGtACR1(+) neurons (left), L5a stGtACR1(–) neurons (middle), and L2/3 stGtACR1(–) neurons (right, *n* = 95). All were from the CFA L2/3 stGtACR1(–) neurons that were imaged above the field of view where the L5a neurons were imaged. ***G***, Bright-field (top) and fluorescent (bottom) images of a representative whole-cell patch-clamped stGtACR1(+) neuron (arrowhead). White lines indicate the location of the patch pipette. Scale bar, 20 µm. ***H***, Representative traces of the membrane potentials of the stGtACR1(+) neurons with and without light illumination. A current of 200 pA was injected into the recorded neuron for 2 s. The orange bar indicates the 594 nm light illumination period of 1 s. ***I***, Normalized spike rates of stGtACR1(+) neurons before and during the light illumination, *p = 1.7 × 10^−6^ by paired *t* test, *n* = 8 neurons from seven slices. ***J***, Bright-field (top) and fluorescent (bottom) images of a representative whole-cell patch-clamped stGtACR1(–) neuron (arrowhead). White lines indicate the location of the patch pipette. Scale bar, 20 µm. ***K***, Representative traces of the membrane potential of the stGtACR1(–) neuron with and without light illumination. A current of 200 pA was injected into the recorded neuron for 2 s. The orange bar indicates the 1 s light illumination period. ***L***, Normalized spike rates of stGtACR1(–) neurons before and during light illumination, *p* = 0.81 by paired *t* test, *n* = 8 neurons from 7 slices.

Finally, to examine the functional contribution of CFA L5a IT neurons to the skilled lever-pull movements, we inactivated CFA L5a IT neurons during lever-pull movement performed after the nontraining days. When *AAV-SIO-stGtACR1-FusionRed* was injected into the left CFA of *Tlx3-Cre* mice, the stGtACR1-FusionRed signal was strongly detected in L5a (Fig. 6*A*), similar to jRGECO1a. To test whether stGtACR1 specifically inhibited the activities of *Tlx3*+ neurons *in vivo*, we injected *AAV-GCaMP6f* together with *AAV-SIO-stGtACR1-FusionRed* into the left CFA of *Tlx3-Cre* mice. This resulted in expression of Cre-independent GCaMP6f signals in CFA (Fig. 6*B*). Then, we simultaneously conducted two-photon imaging and 594 nm light illumination in awake head-fixed mice to evaluate the effect of the light illumination on the spontaneous activity of GCaMP6f-expressing neurons both with and without stGtACR1-FusionRed [with, stGtACR1(+) neurons; without, stGtACR1(−) neurons; Fig. 6*B–E*]. The light illumination substantially reduced the activities of L5a stGtACR1(+) neurons but had no effect on the activities of stGtACR1(−) neurons in L2/3 and L5a (Fig. 6*C–F*). We further conducted whole-cell patch-clamp recording of stGtACR1(+) and stGtACR1(−) neurons in CFA L5a in acute slice preparations. Consistent with the results of *in vivo* two-photon calcium imaging, the activities of stGtACR1(+) neurons were strongly suppressed by the 594 nm light illumination, whereas the activities of stGtACR1(−) neurons remained intact (Fig. 6*G–L*; paired *t* test, *t*_(7)_ = 14.64, *p* = 1.7 × 10^−6^; Fig. 6*I*; *t*_(7)_ = 0.26, *p* = 0.81, Fig. 6*L*). These results indicate that 594 nm light illumination reduced the activities of *Tlx3*+ neurons.

**Figure 7.**
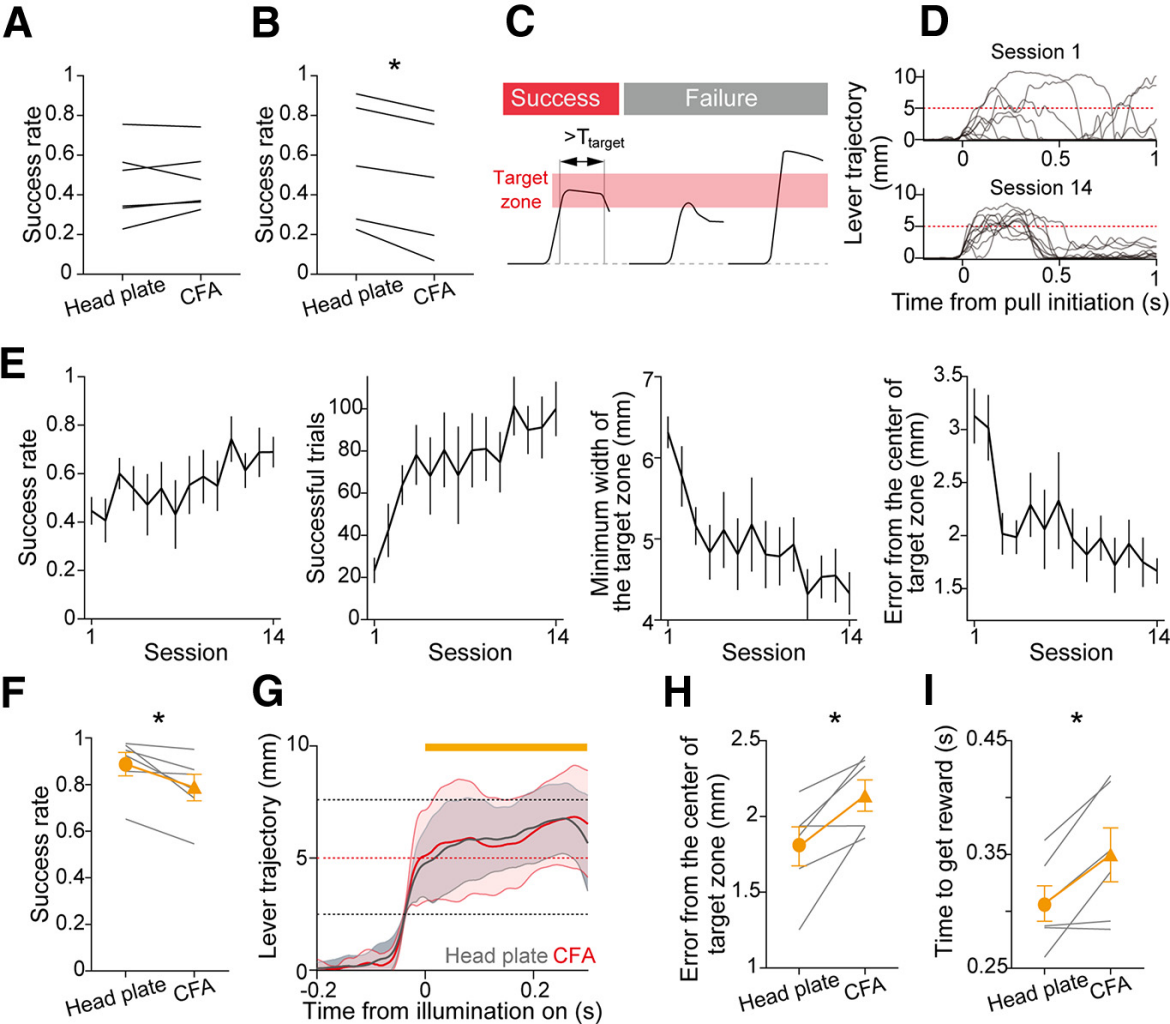
CFA IT neuron-specific optogenetic inactivation during skilled lever pulls. ***A***, Summary of the success rate of the self-initiated lever-pull task in head plate and CFA stimulation trials with the CFA IT-specific optogenetic inactivation at 12 mW, *p* = 0.54 by paired *t* test, *n* = 6 sessions from 5 mice. ***B***, Success rate of the self-initiated lever-pull task with CFA IT neuron-specific optogenetic manipulation under the heavier lever weight (0.05 N), *p = 0.005 by paired *t* test, *n* = 5 sessions from 5 mice. ***C***, The scheme of the target zone lever-pull task. ***D***, Lever trajectories in the first learning session (session 1) and the 14th learning session (session 14) from a representative mouse (*n* = 10 trials in each session). ***E***, Task performance in the target zone lever-pull task in the learning sessions. Left to right, The success rate; the number of successful trials; the minimum width of the target zone; the error from the center of the target zone (the 5 mm pull position from baseline), *n* = 6 mice. ***F***, Summary of the success rate of the target zone lever-pull task in the head plate illumination trials and CFA illumination trials with CFA IT neuron-specific optogenetic manipulation, *p = 0.03 by paired *t* test, *n* = 6 sessions from 6 mice. ***G***, The mean lever trajectories of the target zone lever-pull task in the head plate (gray) illumination trials and CFA illumination (red) trials from a representative mouse; mean ± SD. The orange bar indicates light illumination. ***H***, Summary of the mean errors from the center of the target zone for 0.2 s from initiation in the head plate illumination trials and CFA illumination trials, *p = 0.02 by paired *t* test, *n* = 6 sessions from 6 mice. ***I***, Summary of the time to obtaining reward in the head plate illumination trials and CFA illumination trials, *p = 0.03 by paired *t* test, *n* = 6 sessions from 6 mice.

We then used 594 nm laser light at 12 mW to inhibit the neuronal activities of stGtACR1-expressing *Tlx3*+ neurons in the CFA immediately after the lever-pull initiation. In test sessions, we compared the success rate of trials with laser illumination on the CFA with trials where the illumination was on the head plate. Silencing *Tlx3*+ neurons in CFA immediately after the lever-pull initiation did not affect the success rate (Fig. 7*A*; paired *t* test, *t*_(5)_ = −0.65, *p* = 0.54). We suspected that the lever might have been pulled with a force greater than that required to pull it to its limit (5 mm) and that there would therefore be minimal impact on lever-pull performance, despite a reduction in the activity of L5a IT neurons in the left CFA. Indeed, when we transiently made the lever weight heavier (0.05 N), photoinactivation of *Tlx3*+ neurons in CFA reduced the success rate (Fig. 7*B*; paired *t* test, *t*_(5)_ = 5.59, *p* = 0.005). The DLS, which is downstream of M1 L5a IT neurons, possesses kinematic information (Rueda-Orozco and Robbe, 2015), and DLS inactivation decreases the amplitude of reaches (Lemke et al., 2019) and increases trial-by-trial variability while preserving the sequence structure (Rueda-Orozco and Robbe, 2015). Together, our results raise the possibility that CFA L5a IT neurons might have played a role in the generation of the motor commands that were commensurate with the demands of the skilled movement at the time.

To directly test this possibility, we developed a variation of the lever-pull task in which the mice needed to continue to finely tune the lever position to hold the lever within a certain range of the target position to obtain a reward (target-zone lever-pull task; Fig. 7*C*). The head-fixed mice learned the self-initiated target-zone lever-pull task for 14 learning sessions (Fig. 7*D*,*E*). The range of the target position in which the lever needed to be held (the width of the target zone) was gradually reduced as the learning sessions progressed. The range was set to be approximately ±2 mm from the target center (5 mm from the natural position in the pulling direction) in the 14th session, after the lever trajectories became stereotyped (Fig. 7*D*), and the mean of the absolute distance between the lever position and the target center (the error from the target center) decreased (Fig. 7*E*). Then, we silenced *Tlx3*+ neurons in the left CFA in an optogenetic inactivation session performed after the 14 learning sessions. One-second photoinactivation immediately after the lever-pull initiation caused a significant reduction in the success rate in the CFA illumination trials compared with that in the head plate illumination trials (as the control; Fig. 7*F*; paired *t* test, *t*_(5)_ = 3.09, *p* = 0.03). The error from the target center was significantly larger in the CFA illumination trials than in the head plate illumination trials (Fig. 7*G*,*H*; paired *t* test, *t*_(5)_ = −3.33, *p* = 0.02; Fig. 7*H*), delaying the time from trial initiation at which the mice acquired the water reward (Fig. 7*I*; paired *t* test, *t*_(5)_ = −3.04, *p* = 0.02). These results suggest that CFA L5a IT neurons contributed to moment-to-moment fine adjustment of the skilled lever-pull behavior.

## Discussion

Using *Tlx3-Cre* mice to efficiently access the dynamics of L5a IT neurons, we found that a subset of L5a IT neurons in the CFA (top 20% neurons) retained high prediction accuracy for the lever trajectory in both late learning sessions and sessions performed after 6 d of nontraining. The encoding prediction accuracy of the neuronal ensembles was also stable before and after the nontraining days. Consistent with this, the CFA was necessary even after the nontraining days. By contrast, the motor representation of other CFA L5a IT neurons was not maintained after the nontraining days. Taking into account our previous result (Masamizu et al., 2014), we consider that a subset of CFA L5a IT neurons can become a specific component of the motor memory ensemble through learning and then might be able to remain as a component of the motor memory ensemble for a long period of time, although the nontraining days in our experiment were not of a sufficiently long duration to make comparisons with human motor memory that lasts for years.

We constructed an encoding model with behavioral variables to show that the lever movement that included a major part of the right forelimb movement along the lever-pull axis was more strongly encoded in M1 L5a IT neurons than the other behavioral variables. By contrast, representation of the uninstructed orofacial movements (licking and jaw movements) in individual L5a IT neurons in the CFA was minimal. We also recently revealed that forelimb movement in both self-initiated and external-cue-triggered lever-pull tasks is more strongly encoded in L2/3 neurons in the CFA than are licking and jaw movements (Terada et al., 2022). Thus, the CFA dominantly controls forelimb movement, as suggested by another study (Morandell and Huber, 2017). Licking and jaw movements are probably mainly controlled by another M1 area, the tjM1 (primary tongue-jaw motor cortex; Mercer Lindsay et al., 2019; Terada et al., 2022). Behavioral variables other than lever-related ones also contributed to the activity of M1 L5a IT neurons (Fig. 4*H*). However, compared with the pursued neurons, these variables were not stably represented across the late learning sessions and test sessions (Fig. 5*B*). The session-to-session variability in these movements might be one of the factors influencing the session-to-session variability in neuronal activity (Stringer et al., 2019).

Transient inactivation of CFA L5a IT neurons immediately after the lever-pull initiation in the test session did not decrease the success rate. In the self-initiated lever-pull task, the mice were rewarded if they maintained a pull with more force than the applied magnetic force. Therefore, even if the activity of CFA L5a IT neurons was inhibited, the lever pull would be successful in the case that the reduced output force emitted from the forelimb was greater than the magnetic force, and the lever was not returned on reaching the maximum length of 5 mm. However, in the target-zone lever-pull task in which mice were required to hold the lever in a narrow position, fine adjustment of output force was necessary at any time point. In this case, inactivation of the CFA L5a IT neurons reduced the success rate and increased the difference between the lever and target positions. This suggests that CFA L5a IT neurons would be necessary for the adjustment of the kinematics, which is consistent with previous studies that showed that L5a IT neurons are required for adjustment of the amplitude of reaching in joystick and food-reaching tasks (Park et al., 2022). In the conventional self-initiated lever-pull task, inactivation of the CFA returned the lever to the natural position. In contrast, inactivation of CFA L5a IT neurons in the target-zone lever-pull task did not result in a unidirectional move toward the natural position. Thus, the motor impairment caused by the inactivation of CFA L5a IT neurons might not simply reflect a reduction in the activity of L5a IT neurons to drive L5 pyramidal tract (PT) neurons that innervate the spinal cord (Morishima and Kawaguchi, 2006). PT and IT neurons exhibit different motor-related activity (Turner and DeLong, 2000; Currie et al., 2022; Park et al., 2022), and therefore their functions should be different. For example, inactivation of L5a IT neurons was shown to more severely affect reaching movement than inactivation of L5 PT neurons (Park et al., 2022). We postulated that the motor impairment caused by the photoinactivation of CFA L5a IT neurons in the target-zone lever-pull task might be caused by reduction of the signal transmission from these neurons to the DLS neurons. DLS activity is continuously modulated by kinematic and contextual parameters during sequence execution (Rueda-Orozco and Robbe, 2015), and inactivation of the major output area of the basal ganglia impairs the kinematics (Desmurget and Turner, 2010). This output area projects axons to the ventromedial and ventral anterior motor thalamic nuclei, and these thalamic nuclei project axons to layer 1 in the CFA that come to strongly represent the lever kinematics through learning of the self-initiated lever-pull task (Kuramoto et al., 2009, 2015; Tanaka et al., 2018). This thalamic signal may be updated in the striatum via the corticostriatal inputs from the CFA.

Although inactivation of M1 and the dorsal striatum impaired the gross movement, the effect of inactivation of M1 on skilled fine movement is known to be larger than that from inactivation of striatum (Lemke et al., 2019). Therefore, the final refinement of the forelimb movement may occur in M1. Reorganization of M1 L2/3 neurons is strongly associated with high precision in grasping (Omlor et al., 2019) and context-dependent fine movement proficiency (Terada et al., 2022). Thus, M1 L2/3 neurons might integrate such kinematic signals from the ventromedial and ventral anterior thalamic nuclei with sensory feedback from the sensory area (Mathis et al., 2017) and cerebellum (Dacre et al., 2021) to drive the improvement in fine movement. L2/3 neurons may be necessary for both pull initiation and holding the lever. Furthermore, the corticostriatal inputs may serve as the efference copy carrying the motor kinematics information (Fee, 2014). Although we inferred that the inactivation of CFA L5a IT neurons would severely impair motor kinematics, the effect of such inactivation was significant but small in the target-zone lever-pull task. There are corticostriatal IT neurons in L2/3 and layer 5b (Morishima and Kawaguchi, 2006; Oswald et al., 2013) that may also play roles in motor control. To clarify the role of the corticostriatal IT neurons in the control of the kinematics, it would be useful to record the activity of striatal neurons and retrogradely labeled IT neurons in different layers in the target-zone lever-pull task.

In some motor tasks, M1 L5 PT neurons do not show strong activity in movement initiation, and lesioning of the M1 area and inactivation of M1 L5 PT neurons does not affect well-learned movements (Kawai et al., 2015; Park et al., 2022). In contrast, we previously showed that L5 PT neurons clearly exhibited activity at the initiation of a lever-pull movement and that muscimol injection into the CFA, even after 22–31 sessions, substantially reduced the success rate (Terada et al., 2022). Nonspecific suppression of CFA excitatory neurons immediately after the lever-pull onset deteriorated the lever trajectory with a short latency (∼26 ms in Terada et al., 2022). This short latency suggests direct control of the forelimb movement from the CFA to the spinal cord. Therefore, specific inactivation of PT neurons in the current task may cause more severe deterioration of lever movement than did inactivation of IT neurons. We speculate that skilled forelimb movements may consist of two types, movements that require L5 PT activity and those that do not (Whishaw et al., 1993; Whishaw, 2000; Kawai et al., 2015; Wang et al., 2017). Whether L5 PT neurons are important might depend on what kind of forelimb movement is required for the task (Azim et al., 2014). Long-duration (500 ms) photostimulation of the motor cortex in awake charnnelrhodopsin-2 transgenic mice induces complex ethological movements such as hand-to mouth movement, locomotion, and defensive-like movement with squeaking (Hira et al., 2015). Such ethological movements should be controlled by the brainstem (Roseberry et al., 2016; Capelli et al., 2017; Caggiano et al., 2018). L5 PT neurons and output neurons in the basal ganglia innervate the brainstem nuclei, and the brainstem nuclei also mediate skilled forelimb movements and locomotion (Azim et al., 2014; Esposito et al., 2014; Roseberry et al., 2016; Caggiano et al., 2018; Ferreira-Pinto et al., 2021). If the skilled movement is consolidated in the form of modification of the innate ethological movements, the striatum may be a more dominant control area than M1, whereas if some forelimb movement is learned as a new type of movement, M1 and the corticospinal pathway may be more important (Wang et al., 2017; Ueno et al., 2018; Whishaw, 2000). In both cases, the corticostriatal pathway would be important in kinematic regulation. Cerebellar–thalamic-M1 and cerebellar–spinal pathways are also important for some skilled forelimb movements and locomotion (Low et al., 2018; Tanaka et al., 2018; Sathyamurthy et al., 2020), with the cerebellum and basal ganglia communicating with each other through the pons and thalamus (Bostan and Strick, 2018). In the future, it will be necessary to compare activity and inactivation effects between PT and IT neurons in some same forelimb movement tasks using the same measurement methods and analyses.

## References

[B1] Albarran E, Raissi A, Jáidar O, Shatz CJ, Ding JB (2021) Enhancing motor learning by increasing the stability of newly formed dendritic spines in the motor cortex. Neuron 109:3298–3311.e4. 10.1016/j.neuron.2021.07.030 34437845 PMC8542616

[B2] Aoki S, Smith JB, Li H, Yan X, Igarashi M, Coulon P, Wickens JR, Ruigrok TJ, Jin X (2019) An open cortico-basal ganglia loop allows limbic control over motor output via the nigrothalamic pathway. Elife 8:e49995. 10.7554/eLife.49995 31490123 PMC6731092

[B3] Arthur WJ, Bennett WJ, Stanush PL, McNelly TL (1998) Factors that influence skill decay and retention: a quantitative review and analysis. Hum Perform 11:57–101. 10.1207/s15327043hup1101_3

[B4] Azim E, Jiang J, Alstermark B, Jessell TM (2014) Skilled reaching relies on a V2a propriospinal internal copy circuit. Nature 508:357–363. 10.1038/nature13021 24487617 PMC4230338

[B5] Badreddine N, Zalcman G, Appaix F, Becq G, Tremblay N, Saudou F, Achard S, Fino E (2022) Spatiotemporal reorganization of corticostriatal networks encodes motor skill learning. Cell Rep 39:110623. 10.1016/j.celrep.2022.110623 35385722

[B6] Bostan AC, Strick PL (2018) The basal ganglia and the cerebellum: nodes in an integrated network. Nat Rev Neurosci 19:338–350. 10.1038/s41583-018-0002-7 29643480 PMC6503669

[B7] Caggiano V, Leiras R, Goñi-Erro H, Masini D, Bellardita C, Bouvier J, Caldeira V, Fisone G, Kiehn O (2018) Midbrain circuits that set locomotor speed and gait selection. Nature 553:455–460. 10.1038/nature25448 29342142 PMC5937258

[B8] Callaway EM, et al. (2021) A multimodal cell census and atlas of the mammalian primary motor cortex. Nature 598:86–102. 34616075 10.1038/s41586-021-03950-0 PMC8494634

[B9] Capelli P, Pivetta C, Soledad Esposito M, Arber S (2017) Locomotor speed control circuits in the caudal brainstem. Nature 551:373–377. 10.1038/nature24064 29059682

[B10] Carmena JM, Lebedev MA, Henriquez CS, Nicolelis MA (2005) Stable ensemble performance with single-neuron variability during reaching movements in primates. J Neurosci 25:10712–10716. 10.1523/JNEUROSCI.2772-05.2005 16291944 PMC6725856

[B11] Chestek CA, Batista AP, Santhanam G, Yu BM, Afshar A, Cunningham JP, Gilja V, Ryu SI, Churchland MM, Shenoy KV (2007) Single-neuron stability during repeated reaching in macaque premotor cortex. J Neurosci 27:10742–10750. 10.1523/JNEUROSCI.0959-07.2007 17913908 PMC6672821

[B12] Costa RM, Cohen D, Nicolelis MAL (2004) Differential corticostriatal plasticity during fast and slow motor skill learning in mice. Curr Biol 14:1124–1134. 10.1016/j.cub.2004.06.053 15242609

[B13] Currie SP, Ammer JJ, Premchand B, Dacre J, Wu Y, Eleftheriou C, Colligan M, Clarke T, Mitchell L, Faisal AA, Hennig MH, Duguid I (2022) Movement-specific signaling is differentially distributed across motor cortex layer 5 projection neuron classes. Cell Rep 39:110801. 10.1016/j.celrep.2022.110801 35545038 PMC9620742

[B14] Dacre J, Colligan M, Clarke T, Ammer JJ, Schiemann J, Chamosa-Pino V, Claudi F, Harston JA, Eleftheriou C, Pakan JMP, Huang C-C, Hantman AW, Rochefort NL, Duguid I (2021) A cerebellar-thalamocortical pathway drives behavioral context-dependent movement initiation. Neuron 109:2326–2338.e8. 10.1016/j.neuron.2021.05.016 34146469 PMC8315304

[B15] Dana H, Mohar B, Sun Y, Narayan S, Gordus A, Hasseman JP, Tsegaye G, Holt GT, Hu A, Walpita D, Patel R, Macklin JJ, Bargmann CI, Ahrens MB, Schreiter ER, Jayaraman V, Looger LL, Svoboda K, Kim DS (2016) Sensitive red protein calcium indicators for imaging neural activity. Elife 5:e12727. 10.7554/eLife.12727 27011354 PMC4846379

[B16] Desmurget M, Turner RS (2010) Motor sequences and the basal ganglia: kinematics, not habits. J Neurosci 30:7685–7690. 10.1523/JNEUROSCI.0163-10.2010 20519543 PMC2906391

[B17] Doyon J, Penhune V, Ungerleider LG (2003) Distinct contribution of the cortico-striatal and cortico-cerebellar systems to motor skill learning. Neuropsychologia 41:252–262. 10.1016/s0028-3932(02)00158-6 12457751

[B18] Esposito MS, Capelli P, Arber S (2014) Brainstem nucleus MdV mediates skilled forelimb motor tasks. Nature 508:351–356. 10.1038/nature13023 24487621

[B19] Fee MS (2014) The role of efference copy in striatal learning. Curr Opin Neurobiol 25:194–200. 10.1016/j.conb.2014.01.012 24566242 PMC4153469

[B20] Ferreira-Pinto MJ, Kanodia H, Falasconi A, Sigrist M, Esposito MS, Arber S (2021) Functional diversity for body actions in the mesencephalic locomotor region. Cell 184:4564–4578.e18. 10.1016/j.cell.2021.07.002 34302739 PMC8382160

[B21] Friedrich J, Zhou P, Paninski L (2017) Fast online deconvolution of calcium imaging data. PLoS Comput Biol 13:e1005423. 10.1371/journal.pcbi.1005423 28291787 PMC5370160

[B22] Galea JM, Vazquez A, Pasricha N, Orban de Xivry J-J, Celnik P (2011) Dissociating the roles of the cerebellum and motor cortex during adaptive learning: the motor cortex retains what the cerebellum learns. Cereb Cortex 21:1761–1770. 10.1093/cercor/bhq246 21139077 PMC3138512

[B23] Gerfen CR, Paletzki R, Heintz N (2013) GENSAT BAC Cre-recombinase driver lines to study the functional organization of cerebral cortical and basal ganglia circuits. Neuron 80:1368–1383. 10.1016/j.neuron.2013.10.016 24360541 PMC3872013

[B24] Gerfen CR, Economo MN, Chandrashekar J (2018) Long distance projections of cortical pyramidal neurons. J Neurosci Res 96:1467–1475. 10.1002/jnr.23978 27862192 PMC5429214

[B25] Giovannucci A, Friedrich J, Gunn P, Kalfon J, Brown BL, Koay SA, Taxidis J, Najafi F, Gauthier JL, Zhou P, Khakh BS, Tank DW, Chklovskii DB, Pnevmatikakis EA (2019) CaImAn an open source tool for scalable calcium imaging data analysis. Elife 8:e38173. 10.7554/eLife.38173 30652683 PMC6342523

[B26] Hasan MT, Hernández-González S, Dogbevia G, Treviño M, Bertocchi I, Gruart A, Delgado-García JM (2013) Role of motor cortex NMDA receptors in learning-dependent synaptic plasticity of behaving mice. Nat Commun 4:2258. 10.1038/ncomms3258 23978820 PMC3759079

[B27] Hasegawa R, Ebina T, Tanaka YR, Kobayashi K, Matsuzaki M (2020) Structural dynamics and stability of corticocortical and thalamocortical axon terminals during motor learning. PLoS One 15:e0234930. 10.1371/journal.pone.0234930 32559228 PMC7304593

[B28] Hayashi-Takagi A, Yagishita S, Nakamura M, Shirai F, Wu YI, Loshbaugh AL, Kuhlman B, Hahn KM, Kasai H (2015) Labelling and optical erasure of synaptic memory traces in the motor cortex. Nature 525:333–338. 10.1038/nature15257 26352471 PMC4634641

[B29] Hikosaka O, Nakamura K, Sakai K, Nakahara H (2002a) Central mechanisms of motor skill learning. Curr Opin Neurobiol 12:217–222. 10.1016/s0959-4388(02)00307-0 12015240

[B30] Hikosaka O, Rand M, Nakamura K, Miyachi S, Kitaguchi K, Sakai K, Lu X, Shimo Y (2002b) Long-term retention of motor skill in macaque monkeys and humans. Exp Brain Res 147:494–504. 10.1007/s00221-002-1258-7 12444481

[B31] Hikosaka O, Yamamoto S, Yasuda M, Kim HF (2013) Why skill matters. Trends Cogn Sci 17:434–441. 10.1016/j.tics.2013.07.001 23911579 PMC3756891

[B32] Hira R, Ohkubo F, Ozawa K, Isomura Y, Kitamura K, Kano M, Kasai H, Matsuzaki M (2013) Spatiotemporal dynamics of functional clusters of neurons in the mouse motor cortex during a voluntary movement. J Neurosci 33:1377–1390. 10.1523/JNEUROSCI.2550-12.2013 23345214 PMC6618743

[B33] Hira R, Terada S-I, Kondo M, Matsuzaki M (2015) Distinct functional modules for discrete and rhythmic forelimb movements in the mouse motor cortex. J Neurosci 35:13311–13322. 10.1523/JNEUROSCI.2731-15.2015 26424880 PMC6605479

[B34] Hoens TR, Chawla NV (2013) Imbalanced datasets: from sampling to classifiers. In: Imbalanced learning. (He H, Ma Y, eds), pp 43–59. Hoboken, NJ: Wiley.

[B35] Hwang EJ, Dahlen JE, Hu YY, Aguilar K, Yu B, Mukundan M, Mitani A, Komiyama T (2019) Disengagement of motor cortex from movement control during long-term learning. Sci Adv 5:eaay0001. 10.1126/sciadv.aay0001 31693007 PMC6821459

[B36] Hwang F-J, Roth RH, Wu Y-W, Sun Y, Kwon DK, Liu Y, Ding JB (2022) Motor learning selectively strengthens cortical and striatal synapses of motor engram neurons. Neuron 110:2790–2801.e5. 10.1016/j.neuron.2022.06.006 35809573 PMC9464700

[B37] Im S, Ueta Y, Otsuka T, Morishima M, Youssef M, Hirai Y, Kobayashi K, Kaneko R, Morita K, Kawaguchi Y (2022) Corticocortical innervation subtypes of layer 5 intratelencephalic cells in the murine secondary motor cortex. Cereb Cortex 33:50–67. 10.1093/cercor/bhac052 35396593 PMC9758586

[B38] Jun JJ, et al. (2017) Fully integrated silicon probes for high-density recording of neural activity. Nature 551:232–236. 10.1038/nature24636 29120427 PMC5955206

[B39] Kawai R, Markman T, Poddar R, Ko R, Fantana AL, Dhawale AK, Kampff AR, Ölveczky BP (2015) Motor cortex is required for learning but not for executing a motor skill. Neuron 86:800–812. 10.1016/j.neuron.2015.03.024 25892304 PMC5939934

[B40] Kondo M, Matsuzaki M (2021) Neuronal representations of reward-predicting cues and outcome history with movement in the frontal cortex. Cell Rep 34:108704. 10.1016/j.celrep.2021.108704 33535051

[B41] Kuramoto E, Furuta T, Nakamura KC, Unzai T, Hioki H, Kaneko T (2009) Two types of thalamocortical projections from the motor thalamic nuclei of the rat: a single neuron-tracing study using viral vectors. Cereb Cortex 19:2065–2077. 10.1093/cercor/bhn231 19174446

[B42] Kuramoto E, Ohno S, Furuta T, Unzai T, Tanaka YR, Hioki H, Kaneko T (2015) Ventral medial nucleus neurons send thalamocortical afferents more widely and more preferentially to layer 1 than neurons of the ventral anterior–ventral lateral nuclear complex in the rat. Cereb Cortex 25:221–235. 10.1093/cercor/bht216 23968832

[B43] Lemke SM, Ramanathan DS, Guo L, Won SJ, Ganguly K (2019) Emergent modular neural control drives coordinated motor actions. Nat Neurosci 22:1122–1131. 10.1038/s41593-019-0407-2 31133689 PMC6592763

[B44] Li N, Chen S, Guo ZV, Chen H, Huo Y, Inagaki HK, Chen G, Davis C, Hansel D, Guo C, Svoboda K (2019) Spatiotemporal constraints on optogenetic inactivation in cortical circuits. Elife 8:e48622. 10.7554/eLife.48622 31736463 PMC6892606

[B45] Lopez-Huerta VG, Denton JA, Nakano Y, Jaidar O, Garcia-Munoz M, Arbuthnott GW (2021) Striatal bilateral control of skilled forelimb movement. Cell Rep 34:108651. 10.1016/j.celrep.2020.108651 33472081

[B46] Low AYT, Thanawalla AR, Yip AKK, Kim J, Wong KLL, Tantra M, Augustine GJ, Chen AI (2018) Precision of discrete and rhythmic forelimb movements requires a distinct neuronal subpopulation in the interposed anterior nucleus. Cell Rep 22:2322–2333. 10.1016/j.celrep.2018.02.017 29490269

[B47] Mahn M, Gibor L, Patil P, Cohen-Kashi Malina K, Oring S, Printz Y, Levy R, Lampl I, Yizhar O (2018) High-efficiency optogenetic silencing with soma-targeted anion-conducting channelrhodopsins. Nat Commun 9:4125. 10.1038/s41467-018-06511-8 30297821 PMC6175909

[B48] Masamizu Y, Tanaka YR, Tanaka YH, Hira R, Ohkubo F, Kitamura K, Isomura Y, Okada T, Matsuzaki M (2014) Two distinct layer-specific dynamics of cortical ensembles during learning of a motor task. Nat Neurosci 17:987–994. 10.1038/nn.3739 24880217

[B49] Mathis A, Mamidanna P, Cury KM, Abe T, Murthy VN, Mathis MW, Bethge M (2018) DeepLabCut: markerless pose estimation of user-defined body parts with deep learning. Nat Neurosci 21:1281–1289. 10.1038/s41593-018-0209-y 30127430

[B50] Mathis MW, Mathis A, Uchida N (2017) Somatosensory cortex plays an essential role in forelimb motor adaptation in mice. Neuron 93:1493–1503.e6. 10.1016/j.neuron.2017.02.049 28334611 PMC5491974

[B51] Mercer Lindsay N, Knutsen PM, Lozada AF, Gibbs D, Karten HJ, Kleinfeld D (2019) Orofacial movements involve parallel corticobulbar projections from motor cortex to trigeminal premotor nuclei. Neuron 104:765–780.e3. 10.1016/j.neuron.2019.08.032 31587918 PMC7962749

[B52] Modi MN, Daie K, Turner GC, Podgorski K (2019) Two-photon imaging with silicon photomultipliers. Opt Express 27:35830–35841. 10.1364/OE.27.035830 31878749

[B53] Morandell K, Huber D (2017) The role of forelimb motor cortex areas in goal directed action in mice. Sci Rep 7:15759. 10.1038/s41598-017-15835-2 29150620 PMC5693936

[B54] Morishima M, Kawaguchi Y (2006) Recurrent connection patterns of corticostriatal pyramidal cells in frontal cortex. J Neurosci 26:4394–4405. 10.1523/JNEUROSCI.0252-06.2006 16624959 PMC6674016

[B55] Muñoz-Castañeda R, et al. (2021) Cellular anatomy of the mouse primary motor cortex. Nature 598:159–166. 10.1038/s41586-021-03970-w 34616071 PMC8494646

[B56] Musall S, Kaufman MT, Juavinett AL, Gluf S, Churchland AK (2019) Single-trial neural dynamics are dominated by richly varied movements. Nat Neurosci 22:1677–1686. 10.1038/s41593-019-0502-4 31551604 PMC6768091

[B57] Omlor W, Wahl A-S, Sipilä P, Lütcke H, Laurenczy B, Chen I-W, Sumanovski LT, van 't Hoff M, Bethge P, Voigt FF, Schwab ME, Helmchen F (2019) Context-dependent limb movement encoding in neuronal populations of motor cortex. Nat Commun 10:4812. 10.1038/s41467-019-12670-z 31645554 PMC6811620

[B58] Orban de Xivry J-J, Criscimagna-Hemminger SE, Shadmehr R (2011) Contributions of the motor cortex to adaptive control of reaching depend on the perturbation schedule. Cereb Cortex 21:1475–1484. 10.1093/cercor/bhq192 21131448 PMC3116732

[B59] Oswald MJ, Tantirigama ML, Sonntag I, Hughes SM, Empson RM (2013) Diversity of layer 5 projection neurons in the mouse motor cortex. Front Cell Neurosci 7:174. 10.3389/fncel.2013.00174 24137110 PMC3797544

[B60] Pachitariu M, Sridhar S, Stringer C (2023) Solving the spike sorting problem with Kilosort. bioRxiv 523036. 10.1101/2023.01.07.523036.

[B61] Park J, Phillips JW, Guo JZ, Martin KA, Hantman AW, Dudman JT (2022) Motor cortical output for skilled forelimb movement is selectively distributed across projection neuron classes. Sci Adv 8:eabj5167. 10.1126/sciadv.abj5167 35263129 PMC8906739

[B62] Penhune VB, Doyon J (2002) Dynamic cortical and subcortical networks in learning and delayed recall of timed motor sequences. J Neurosci 22:1397–1406. 10.1523/JNEUROSCI.22-04-01397.2002 11850466 PMC6757579

[B63] Peters AJ, Chen SX, Komiyama T (2014) Emergence of reproducible spatiotemporal activity during motor learning. Nature 510:263–267. 10.1038/nature13235 24805237

[B64] Roseberry TK, Lee AM, Lalive AL, Wilbrecht L, Bonci A, Kreitzer AC (2016) Cell-type-specific control of brainstem locomotor circuits by basal ganglia. Cell 164:526–537. 10.1016/j.cell.2015.12.037 26824660 PMC4733247

[B65] Rueda-Orozco PE, Robbe D (2015) The striatum multiplexes contextual and kinematic information to constrain motor habits execution. Nat Neurosci 18:453–460. 10.1038/nn.3924 25622144 PMC4342106

[B66] Runyan CA, Piasini E, Panzeri S, Harvey CD (2017) Distinct timescales of population coding across cortex. Nature 548:92–96. 10.1038/nature23020 28723889 PMC5859334

[B67] Santos FJ, Oliveira RF, Jin X, Costa RM (2015) Corticostriatal dynamics encode the refinement of specific behavioral variability during skill learning. Elife 4:e09423. 10.7554/eLife.09423 26417950 PMC4616249

[B68] Sathyamurthy A, Barik A, Dobrott CI, Matson KJE, Stoica S, Pursley R, Chesler AT, Levine AJ (2020) Cerebellospinal neurons regulate motor performance and motor learning. Cell Rep 31:107595. 10.1016/j.celrep.2020.107595 32402292 PMC7263484

[B69] Sauerbrei BA, Guo J-Z, Cohen JD, Mischiati M, Guo W, Kabra M, Verma N, Mensh B, Branson K, Hantman AW (2020) Cortical pattern generation during dexterous movement is input-driven. Nature 577:386–391. 10.1038/s41586-019-1869-9 31875851 PMC6962553

[B70] Shamash P, Carandini M, Harris K, Steinmetz N (2018) A tool for analyzing electrode tracks from slice histology. bioRxiv 447995. 10.1101/447995.

[B71] Smith KS, Graybiel AM (2013) A dual operator view of habitual behavior reflecting cortical and striatal dynamics. Neuron 79:361–374. 10.1016/j.neuron.2013.05.038 23810540 PMC3951965

[B72] Stringer C, Pachitariu M, Steinmetz N, Reddy CB, Carandini M, Harris KD (2019) Spontaneous behaviors drive multidimensional, brainwide activity. Science 364:eaav7893. 10.1126/science.aav7893 31000656 PMC6525101

[B73] Tanaka YH, Tanaka YR, Furuta T, Yanagawa Y, Kaneko T (2008) The effects of cutting solutions on the viability of GABAergic interneurons in cerebral cortical slices of adult mice. J Neurosci Methods 171:118–125. 10.1016/j.jneumeth.2008.02.021 18430473

[B74] Tanaka YH, Tanaka YR, Kondo M, Terada S-I, Kawaguchi Y, Matsuzaki M (2018) Thalamocortical axonal activity in motor cortex exhibits layer-specific dynamics during motor learning. Neuron 100:244–258.e12. 10.1016/j.neuron.2018.08.016 30174116

[B75] Terada S-I, Kobayashi K, Matsuzaki M (2022) Transition of distinct context-dependent ensembles from secondary to primary motor cortex in skilled motor performance. Cell Rep 41:111494. 10.1016/j.celrep.2022.111494 36260994

[B76] Turner RS, DeLong MR (2000) Corticostriatal activity in primary motor cortex of the macaque. J Neurosci 20:7096–7108. 10.1523/JNEUROSCI.20-18-07096.2000 10995857 PMC6772845

[B77] Ueno M, Nakamura Y, Li J, Gu Z, Niehaus J, Maezawa M, Crone SA, Goulding M, Baccei ML, Yoshida Y (2018) Corticospinal circuits from the sensory and motor cortices differentially regulate skilled movements through distinct spinal interneurons. Cell Rep 23:1286–1300.e7. 10.1016/j.celrep.2018.03.137 29719245 PMC6608728

[B78] Wang X, Liu Y, Li X, Zhang Z, Yang H, Zhang Y, Williams PR, Alwahab NSA, Kapur K, Yu B, Zhang Y, Chen M, Ding H, Gerfen CR, Wang KH, He Z (2017) Deconstruction of corticospinal circuits for goal-directed motor skills. Cell 171:440–455.e14. 10.1016/j.cell.2017.08.014 28942925 PMC5679421

[B79] Whishaw IQ (2000) Loss of the innate cortical engram for action patterns used in skilled reaching and the development of behavioral compensation following motor cortex lesions in the rat. Neuropharmacology 39:788–805. 10.1016/s0028-3908(99)00259-2 10699445

[B80] Whishaw IQ, Pellis SM, Gorny B, Kolb B, Tetzlaff W (1993) Proximal and distal impairments in rat forelimb use in reaching follow unilateral pyramidal tract lesions. Behav Brain Res 56:59–76. 10.1016/0166-4328(93)90022-i 7691077

[B81] Winnubst J, et al. (2019) Reconstruction of 1,000 projection neurons reveals new cell types and organization of long-range connectivity in the mouse brain. Cell 179:268–281.e13. 10.1016/j.cell.2019.07.042 31495573 PMC6754285

[B82] Xu T, Yu X, Perlik AJ, Tobin WF, Zweig JA, Tennant K, Jones T, Zuo Y (2009) Rapid formation and selective stabilization of synapses for enduring motor memories. Nature 462:915–919. 10.1038/nature08389 19946267 PMC2844762

[B83] Yamamoto R, Furuyama T, Sugai T, Ono M, Pare D, Kato N (2020) Serotonergic control of GABAergic inhibition in the lateral amygdala. J Neurophysiol 123:670–681. 10.1152/jn.00500.2019 31875487 PMC7395475

[B84] Yin HH, Mulcare SP, Hilário MRF, Clouse E, Holloway T, Davis MI, Hansson AC, Lovinger DM, Costa RM (2009) Dynamic reorganization of striatal circuits during the acquisition and consolidation of a skill. Nat Neurosci 12:333–341. 10.1038/nn.2261 19198605 PMC2774785

[B85] Yizhar O, Fenno LE, Davidson TJ, Mogri M, Deisseroth K (2011) Optogenetics in neural systems. Neuron 71:9–34. 10.1016/j.neuron.2011.06.004 21745635

[B86] Zuo Y, Yang G, Kwon E, Gan W-B (2005) Long-term sensory deprivation prevents dendritic spine loss in primary somatosensory cortex. Nature 436:261–265. 10.1038/nature03715 16015331

